# Non-pathogenic Trojan horse *Nissle1917* triggers mitophagy through PINK1/Parkin pathway to discourage colon cancer

**DOI:** 10.1016/j.mtbio.2024.101273

**Published:** 2024-09-27

**Authors:** Yang Wang, Yao Liu, Xiaomin Su, Lili Niu, Nannan Li, Ce Xu, Zanya Sun, Huishu Guo, Shun Shen, Minghua Yu

**Affiliations:** a Pharmacy Department, Shanghai Pudong Hospital, Fudan University Pudong Medical Center, Shanghai, 201399, China; b Central Laboratory, First Affiliated Hospital, Institute (college) of Integrative Medicine, Dalian Medical University, Dalian, 116011, China; c Clinical Oncology Center, Shanghai Municipal Hospital of TCM, Shanghai University of Traditional Chinese Medicine, Shanghai, 200071, China; d Fudan University Clinical Research Center for Cell-based Immunotherapy & Department of Oncology, Fudan University Pudong Medical Center, Shanghai, 201399, China

**Keywords:** RBC membrane, *EcN*@RBC, Mitophagy, Apoptosis

## Abstract

Bacteria-mediated antitumor therapy has gained widespread attention for its innate tumor-targeting capability and excellent immune activation properties. Nevertheless, the clinical approval of bacterial therapies remains elusive primarily due to the formidable challenge of balancing safety with enhancing *in vivo* efficacy. In this study, leveraging the probiotic *Escherichia coli Nissle1917* (*EcN*) emerges as a promising approach for colon cancer therapy, offering a high level of safety attributed to its lack of virulence factors and its tumor-targeting potential owing to its obligate anaerobic nature. Specifically, we delineate the erythrocyte (RBC) membrane-camouflaged *EcN*, termed as Trojan horse *EcN*@RBC, which triggers apoptosis in tumor cells by mitigating mitochondrial membrane potential (MMP) and subsequently activating the PINK1/Parkin pathway associated with mitophagy. Concurrently, the decline in MMP induced by mitophagy disrupts the mitochondrial permeability transition pore (MPTP), leading to the release of Cytochrome C and subsequent apoptosis induction. Moreover, synergistic effects were observed through the combination of the autophagy activator rapamycin, bolstering the antitumor efficacy *in vivo*. These findings offer novel insights into probiotic-mediated antitumor mechanisms and underscore the therapeutic potential of *EcN*@RBC for colon cancer patients.

## Graphical abstract

**Figure:**
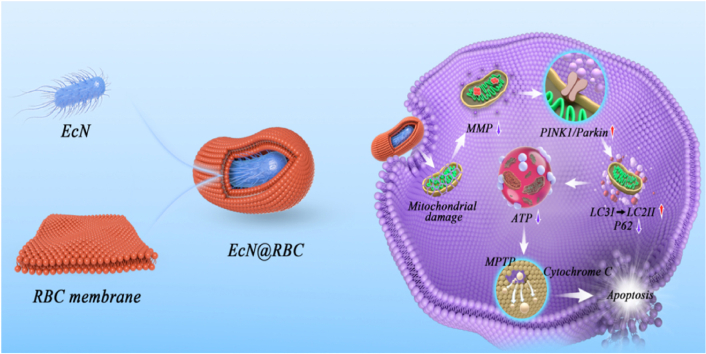

## 1 Introduction

Microbes constitute essential components of ecosystems and play a vital role in maintaining mammalian homeostasis and host health. Various microbes have been employed for anti-tumor therapy due to their unique ability to target tumors and activate natural immune responses [[1], [2], [3]]. It has been reported that some attenuated immunogenic engineered bacteria have been applied to anti-tumor therapy [4]. *Salmonella VNP20009*, which has shown promising results in phase I clinical trials due to its tumor targeting and killing ability [5,6]. Recombinant *Listeria monocytogene* is mainly widely used in tumor immunotherapy, leveraging its ability to intracellularly replicate within host phagocytes to enhance specific immune responses within the tumor microenvironment, disrupt immune tolerance, and provoke robust and enduring immune reactions from antigen-specific CD8^+^ T cells [7,8]. Despite modifications to reduce virulence, attenuated immunogenic bacteria still pose inherent risks, notably due to their rapid multiplication capacity and inevitable immunogenicity, constraining their clinical utility. Moreover, there exists a significant challenge in improving safety while concurrently augmenting clinical efficacy.

*Escherichia coli Nissle1917* (*EcN*), a commonly utilized probiotic, has been extensively studied across various disease research known for its low toxicity and amenability to genetic manipulation [[9], [10], [11]]. *EcN* can enhance host immune response to tumors by modulating the intestinal microenvironment and immune cell activity [12]. This immunostimulatory effect boosts immune cell activity, thereby suppressing tumor cell growth and spread [13]. Studies have shown that by utilizing a stable lysis release mechanism, *EcN* was engineered as a nanobody capable of controlling the production and release of PD-L1 and cytotoxic T-lymphocyte-associated protein 4 (CTLA-4) [14]. It has also been shown that *EcN* is designed to sense blue light and release encoded flagellin B (flaB), which is conjugated with lanthanide-doped upconversion nanoparticles (UCNPs) for near-infrared (NIR) nanogenetic cancer immunotherapy. Under 808 nm light irradiation, UCNPs emit blue light, triggering *EcN* to secrete flaB, which subsequently binds to toll-like receptor 5 expressed on macrophage membranes via a MyD88-dependent signaling pathway to activate immune responses [15]. Furthermore, the probiotic *EcN* has been genetically engineered to express secreted interleukin-2 (IL-2), which has excellent biocompatibility and efficiency in regulating intestinal flora and intestinal inflammation [16]. It has been reported that combination of *EcN* with Galunisertib exerts antitumor efficacy by augmenting tumor-specific effector T cell infiltration and dendritic cell activation [17]. Clinical trials have shown that *EcN* enrichment in tumor samples of colon cancer patients exceeded that in normal tissues following *EcN* administration. Genetically engineered *EcN* holds promise as an oral platform for disease detection and treatment in colon cancer through the generation of screening and therapeutic molecules [18]. *EcN* not only orchestrates host immune regulation and maintains equilibrium in immune factor secretion but also regulates antimicrobial peptide expression, enhances the secretion of immunoglobulin A (IgA), and fosters anti-inflammatory immune responses by upregulating mucin production [19]. Therefore, *EcN* exhibits high safety and tolerability. Nonetheless, the therapeutic efficacy of *EcN* in clinical applications is hampered by an unclear mechanistic understanding of its actions.

Multiple studies have demonstrated that bacteria primarily facilitate anti-tumor therapy through surface proteins or metabolite to induce apoptosis, pyroptosis, and autophagy [20]. The precise role of autophagy in cancer, whether as a tumor-suppressor or tumor-promoter remains to be definitively determined [[21], [22], [23]]. Autophagy, a degradation process that transports nucleic acids, proteins, and organelles to lysosomes, can selectively clear specific organelles as well [24]. Bioactive substances secreted by probiotics and symbiotic bacteria have been shown to induce autophagy in intestinal epithelial cells, potentially contributing to the beneficial clinical effects associated with healthy gut microbiota and probiotic interventions [25]. While autophagy may enhance stress tolerance and the survival of tumor cells, excessive autophagy can impede tumor progression and metastasis at various stages and may even induce tumor cell death [23,26]. Some bacteria have evolved multiple strategies to manipulate the autophagy signaling pathway or hinder the fusion of autophagosomes with lysosomes, thus evading autophagic degradation and exploiting autophagy to promote their own growth and proliferation [[27], [28], [29], [30]]. For instance, *Lactobacillus rhamnosus LGR-1* inhibited the activation of ROS and NLRP3 inflammasome and prevented *Escherichia coli* induced apoptosis through PINK1/Parkin-mediated mitophagy [[31], [32], [33]]. *Bacillus SC06* activated p38 MAPK signaling pathway, down-regulated AKT, reduced ROS production and induced autophagy by regulating the expression of apoptotic protein Bcl-2, Bax, and caspase-3 [34,35]. Mitochondria, highly developed organelles, balance energy demand and supply by consuming substantial amounts of ATP. During transitions from aerobic to anaerobic states, mitophagy decreases mitochondrial numbers, enabling cells to adapt to anaerobic condition [[36], [37], [38]]. This form of autophagy involves the delivery of damaged mitochondria to lysosomes for degradation, favoring the maintenance of mitochondrial quality and quantity across different cell types [[39], [40], [41]]. When mitochondria sustains significant damage, cells initiate apoptosis or autophagic death programs to eliminate these impaired organelles [33,42]. Furthermore, several mitochondria-targeting drugs have been found to stimulate excessive mitophagy and inhibit the progression of colon cancer. For instance, 3-Carboxyl proxyl nitroxide (Mito-CP) and mitochondrial metformin, two mitochondria-targeting compounds, released UNC-51-like autophagy activating kinase 1 (ULK1) from mTOR-mediated inhibition, affecting mitochondrial morphology and reduced mitochondrial membrane potential to produce mitophagy. Which providing an attractive approach for colon cancer intervention [43]. Additionally, The Mitochondrial Disruptor Devimistat (CPI-613), by disrupting mitochondrial membrane potential, induces cell death, synergizing with the anti-tumor drugs 5-FU and irinotecan to inhibit colon cancer development [44]. Although the precise therapeutic mechanism warrants further exploration. Autophagy exerts a bidirectional regulatory effect and dynamically influences tumor growth promotion or inhibition at various stages of tumorigenesis, indicating its potential as an intervention strategy for cancer. There are also reports that *EcN* can affect mitochondrial function, but the specific underlying mechanism behind this still needs further exploration [45,46].

Nanoparticle-based drug delivery systems have historically been employed in both clinical and experimental settings to enhance drug efficacy. Notably, erythrocyte membrane (RBC)-coated nanoparticles have shown significant success in anti-tumor research [47]. The extended circulation of RBC is facilitated by various membrane proteins, including CD47, a protein with five transmembrane domains that acts as a self-marking protein [48]. CD47 interacts with signal regulatory protein alpha (SIRPα) on macrophages, delivering a “don't eat me” signal and thereby preventing phagocytosis by immune cells. Additionally, erythrocytes, which are the most abundant cells in the body, have a circulation time of 120 days, substantially longer than PEG-modified nanoparticles, which circulate for approximately 15.8 h [49]. By modifying the surface or conjugating targeting ligands, RBCs can also bind to specific receptors or molecular markers on tumor cells, enabling precise drug release and targeted therapy [50,51]. The combined benefits of prolonged circulation and tumor-targeting capabilities make RBC-based Trojan horse drug delivery systems a promising therapeutic strategy and a widely explored drug delivery platform.

**Scheme 1:**
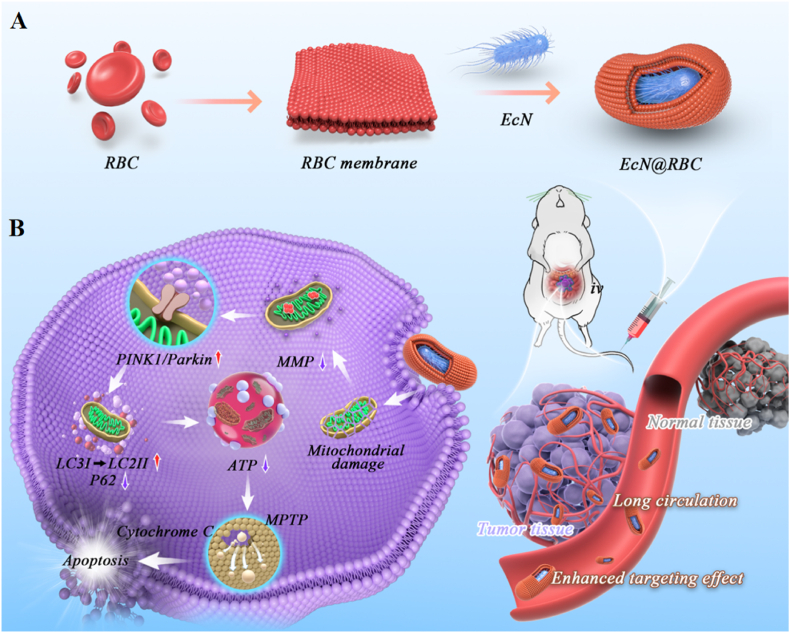
Schematic of *EcN*@RBC preparation and anti-tumor mechanism. (A) The extraction of RBC membrane and the preparation of *EcN*@RBC. (B) The anti-tumor mechanism of *EcN*@RBC.

Herein, we synthesized the *EcN*@RBC by integrating the RBC membrane onto the surface of *EcN* [52] (Scheme 1). The RBC membrane not only effectively mitigated the release of toxins, but also acts as a Trojan horse by prolonging the retention of bacteria in the bloodstream and enhancing tumor enrichment. In addition, we found that *EcN*@RBC induced mitochondrial damage by decreasing mitochondrial membrane potential (MMP). As MMP decreased, PINK1/Parkin signaling was activated to mediate mitophagy. Activated Parkin proteins ubiquitinated various mitochondrial outer membrane proteins, forming complexes with autophagy adaptor protein P62. This complex facilitated mitophagy by binding with LC3, contributing to cellular energy insufficiency, increased permeability of the mitochondrial permeability transition pore (MPTP), and release of Cytochrome C (Cyto-C), thereby inducing apoptosis in colon cancer cells. Furthermore, combining *EcN*@RBC with Rapamycin enhanced the anti-tumor effect *in vivo*. These findings present novel potential strategies for probiotic-mediated antitumor therapy, suggesting further exploration of the regulatory mechanisms of autophagy to develop more effective treatment modalities for cancer patients.

## 2 Results and discussion

### 2.1 Preparation and characterization of *EcN*@RBC

**Fig. 1:**
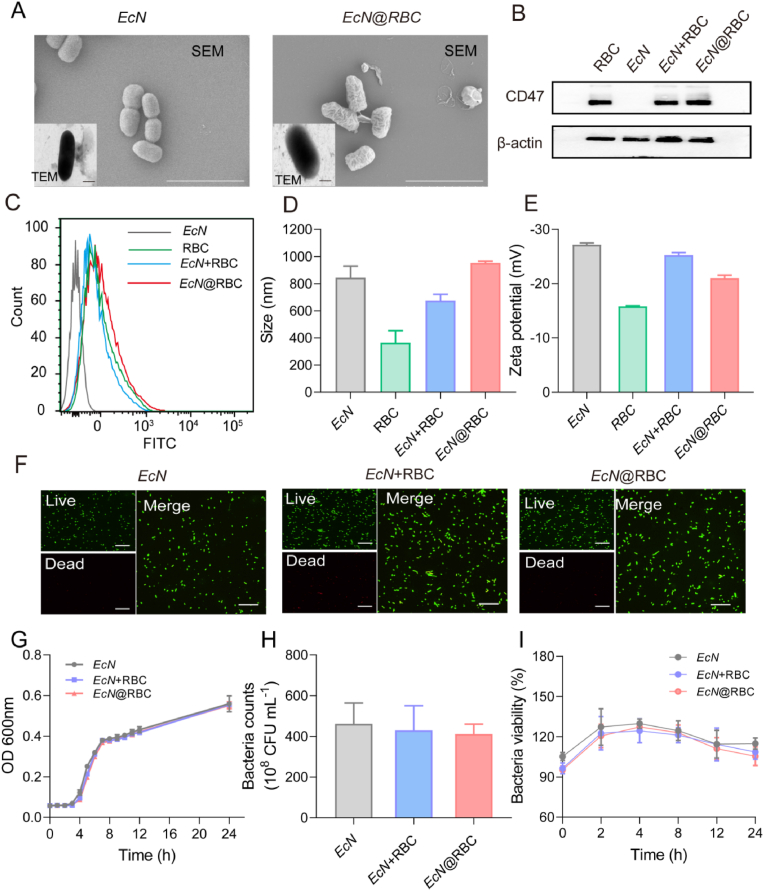
Preparation and characterization of *EcN*@RBC. (A) Representative TEM (Scale bar = 0.5 μm) and SEM (Scale bar = 3 μm) images of *EcN* and *EcN*@RBC. (B) The expression level of CD47 in different group. (C) Flow cytometric analysis of *EcN*, RBC, *EcN* + RBC, and *EcN*@RBC (*EcN*, 1 × 10^6^ CFU/mL). (D) The hydrodynamic diameters and (E) zeta potentials of each group. (F) Visualization of dead and live bacteria after incubation for 12 h. Green fluorescence represents living bacteria, red fluorescence represents dead bacteria (Scale bar = 10 μm). (G) The growth curves of each group at specified time points. (H) The enumeration of colonies of *EcN*, *EcN* + RBC, and *EcN*@RBC after incubation for 24 h (*EcN*, 1 × 10^6^ CFU/mL). (I) Cell viability analysis of *EcN*, *EcN* + RBC, and *EcN*@RBC by AlamarBlue.

*EcN*, as a special probiotic, not only colonizes the intestinal tract and prevents the invasion of the intestinal mucosa by pathogenic bacteria, thereby safeguarding the intestinal barrier, but also accurately delivers anticancer proteins to the tumor site and infiltrate tumor cells [[14], [15], [16]]. Leveraging these properties, *EcN* has been extensively applied in the treatment of inflammatory bowel disease and solid tumors [[53], [54], [55]]. The red blood cell membrane delivery system has been widely used in anti-tumor research, boasting distinctive characteristics such as biocompatibility, low immunogenicity, and prolonged circulation time, which facilitate more effective drug delivery to the target site. The encapsulation method employed in our study is well-established and widely recognized in the literature [52]. Specifically, red blood cell (RBC) membranes were extracted from male Balb/c mice aged 6–8 weeks. These RBC membranes were mixed with *EcN* and subsequently extruded through a porous polycarbonate membrane with a pore size of 1 μm. Subsequently, we characterized *EcN*@RBC using scanning electron microscopy (SEM) and transmission electron microscopy (TEM), with the results presented in Fig. 1A. The TEM images showed that the surfaces of uncoated *EcN* are smooth with well-defined edges. In contrast, *EcN*@RBC exhibits a transparent phospholipid bilayer shell on its surface, which may be attributed to the irregular coating formed by the RBC membrane. Similarly, the SEM images revealed that the surface of *EcN*@RBC is rough due to the RBC membrane coating, whereas the uncoated *EcN* maintains a smooth surface, consistent with the natural morphology of the bacteria. *EcN*@RBC was further visualised by laser scanning confocal microscopy (LSCM). The LSCM image shown in Fig. S1A revealed that there was no significant colocalization of red and blue fluorescence in the *EcN* + RBC group, while the fluorescence colocalization was stronger in the *EcN*@RBC group. In addition, various marker proteins adorn the surface of the RBC membrane, with CD47 serving as a pivotal marker for autoreactive immune cells to recognize RBC [56]. It plays a crucial role in maintaining the morphology of RBC and preventing the attacks of autoimmune cells. To validate the success of the coating encapsulation, *EcN*, RBC membranes with physically mixed *EcN* (*EcN* + RBC) and RBC membranes with extruded *EcN* (*EcN*@RBC) groups were detected by western blotting, confirming the expression of CD47 protein in *EcN*@RBC group (Fig. 1B). To further characterize the encapsulation of *EcN* by the RBC membrane, we labeled CD47 on the RBC membrane with green fluorescence and *EcN* with blue fluorescence. As shown in the Fig. S1B, laser CLSM revealed no colocalization of green and blue fluorescence in the *EcN* + RBC group. In contrast, the *EcN*@RBC group exhibited clear colocalization of green and blue fluorescence. Additionally, flow cytometry showed a peak shift in *EcN*@RBC group, indicating the successful coating of the RBC membrane (Fig. 1C). Furthermore, the particle size and potential of each group were detected, revealing that the *EcN* initially sized at 800 nm, uniformly increased by 200 nm after coating the RBC membrane (Fig. 1D). The surface ζ potential of *EcN*@RBC increased approximately 8 mV compared with *EcN* (Fig. 1E), possibly due to the partial charge coverage of *EcN* by the RBC membrane, resulting in a decrease in the absolute value of the potential. This result probably resulted from the successful wrapping of *EcN* around the erythrocyte membrane to form the Trojan horse *EcN*@RBC.

Subsequently, to ascertain whether the RBC membrane impacts the growth of *EcN*, *EcN*, *EcN* + RBC, and *EcN*@RBC groups were stained by Live/Dead cell double staining reagents. The observation results under LSCM were instructed in Fig. 1F. The green fluorescence intensity in the *EcN*, *EcN* + RBC, and *EcN*@RBC groups remained relatively consistent, indicating that bacteria could thrive with RBC membrane, and the influence of membrane coating on bacterial viability could be considered negligible. Additionally, the growth curve of *EcN*@RBC was recorded to investigate whether the RBC membrane affected the viability of bacteria. As shown in Fig. 1G, the *EcN*, *EcN* + RBC, and *EcN*@RBC groups exhibited similar rates and reached the same optical density within 24 h. Meanwhile, there was no significant difference in colony the numbers among the *EcN*, *EcN* + RBC, and *EcN*@RBC groups after 24 h (Fig. 1H and Fig. S2). Furthermore, the AlamarBlue was used to assess bacterial viability, with results indicating no significant difference in livability among *EcN*, *EcN* + RBC, and *EcN*@RBC groups (Fig. 1I). In general, the *EcN*@RBC was successfully prepared and its functionality remained unaffected by the membrane coating, laying the groundwork for subsequent research.

### 2.2 Study on the biological safety, blood retention, and biodistribution of *EcN*@RBC *in vivo*

**Fig. 2:**
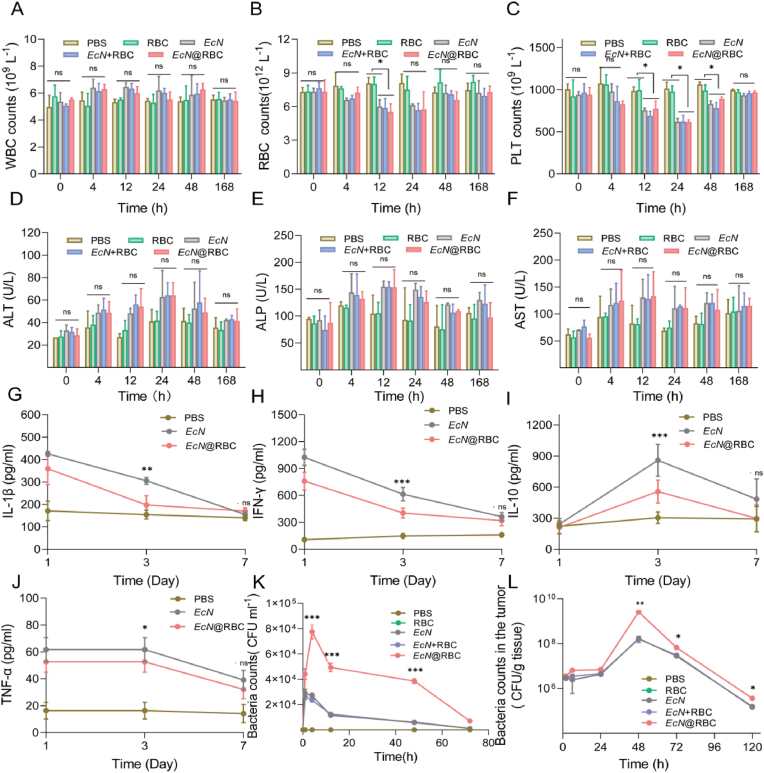
Assessment of biological safety, blood retention, and biodistribution *in vivo*. (A–C) The routine blood analysis including WBC, RBC, and PLT counts (n = 6). Reference range of WBC count: 0.8–6.8 10^9^/L; RBC count: 6.36–9.42 101 [2]/L; PLT count: 450–1590 10^9^/L. (D–F) The levels of ALT, ALP, and AST levels. Reference range of ALP: 22.52–474.35 U/L; ALT: 10.06–96.47 U/L; AST: 36.31–235.48 U/L (n = 6). The level of inflammatory factor *in vivo*. The expression levels of cytokines (G) IL-β, (H) IFN-γ, (I) IL-10, and (J) TNF-α in serums (n = 6). (K) The distribution of *EcN* in the blood of different groups. (L) The relationship between time after administration intravenously and *EcN* number within tumor (n = 3). Significance was assessed using student's t-test, giving p values, ∗p < 0.05, ∗∗p < 0.01, ∗∗∗p < 0.005, ns indicates no statistical significance.

Ensuring the safety of bacteria, particularly with regards to *EcN*@RBC, is our primary concern, thus prompting an investigation into its biosafety in organisms. The blood routine and cytokines were employed to detect the inflammatory response induced by *EcN*@RBC, both of which are commonly utilized to assess the side effects of bacterial therapy. The level of white blood cells (WBC), red blood cells (RBC), platelets (PLT), alanine phosphatases (ALP), alkaline aminotransferase (ALT) and aspartate transaminase (AST) were shown in Fig. 2A–F. There was no remarkable difference between the RBC group and PBS group. Remarkably, the levels of RBC and PLT were diminished in *EcN*, *EcN* + RBC, and *EcN*@RBC group compared to PBS group after 12 h of administration. but returned to baseline levels comparable to PBS by 168 h. Moreover, the pertinent liver function indices in the *EcN*, *EcN* + RBC, and *EcN*@RBC groups exhibited no significant alterations when compared with those in the PBS group, all remaining within normal ranges within 7 days post-administration. This suggested that the acute inflammation induced by *EcN*, *EcN* + RBC, and *EcN*@RBC was mild and well-tolerated, with resolution occurring within one week without the manifestation of chronic toxicity. To further assess alterations in inflammation levels, enzyme-linked immunosorbent assay (ELISA) kits were employed to measure cytokine levels in the sera of the respective groups at various time points, with the results depicted in Fig. 2G–J. The levels of cytokines such as interleukin-β (IL-β), interferon-γ (IFN-γ), interleukin-10 (IL-10), and tumor necrosis factor-α (TNF-α) in both the *EcN* group and the *EcN*@RBC group were higher than those in the PBS group. At the third day, the levels of inflammatory cytokines in the *EcN* group were dramatically improved compared with those in the *EcN*@RBC. Fortunately, the cytokine levels in each group returned to normal within a week, indicating that the RBC membrane had favorable biosafety. This confirmed that RBC encapsulation of *EcN* reduces the bodily clearance of bacteria due to the anti-phagocytic nature of RBC and also diminishes the inflammatory response caused by bacteria.

Subsequently, the blood preservation of *EcN in vivo* was investigated by intravenous administration of different reagents. Blood samples were collected from mice in each at various time points, diluted and spread on LB agar plate, as shown in Fig. S3. Although both *EcN* and *EcN* + RBC group were rapidly cleared from the bloodstream after intravenous administration, the residual amount of *EcN* in the blood of mice treated with *EcN*@RBC was significantly higher compared to that in the *EcN* and *EcN* + RBC groups. Specifically, the content of *EcN* in the blood of mice treated with *EcN*@RBC was approximately 3, 4, and 7 times higher than that in mice treated with *EcN* at 4 h, 12 h, and 48 h after intravenous administration, respectively (Fig. 2K). This result indicated that the membrane-coated *EcN* significantly increased its retention in the blood. For a long time, the accumulation of drugs in tumor tissue has played a crucial role in cancer treatment. After confirming the improvement of blood retention and biocompatibility with *EcN*@RBC, the orthotopic colon cancer tumor model was established to evaluate the colonization of *EcN* in tumor and to circumvent immune responses associated with different blood types. Balb/c mice were selected for the biodistribution study [57]. Following intravenous injection of the respective groups, lung, liver, spleen, kidney, heart, and tumor tissues were collected at different time points. After dilution and plating on LB agar plates, the number of bacteria in each tissue was assessed to appraise their biodistribution. As anticipated, the level of *EcN* in the liver and spleen were markedly enriched in the *EcN*@RBC group within 2 h after intravenous administration (Fig. S4). Furthermore, the level of *EcN* in tumor tissue dramatically increased within 2 days after intravenous administration, with the number of bacteria in tumor was 100 times higher than in healthy tissue (Fig. 2L). The tumor tissues were heavily colonized by the bacteria, which gradually decreased over time. The high aggregation of these bacteria in tumors might be attributed to their preference for the hypoxic environment of the tumor [58]. We hypothesized that the increased accumulation of bacteria in tumor tissues of mice injected with *EcN*@RBC was likely due to their prolonged retention in the bloodstream, providing greater opportunities for bacterial colonization in the tumor. Moreover, the levels of *EcN* in the lung, liver, spleen, kidney, and heart of mice intravenously injected with *EcN*@RBC were significantly lower than those of mice injected with *EcN* (Fig. S5). This phenomenon could be attributed to the presence of RBC membranes reducing cellular uptake. These data indicated that bacterial encapsulation by RBC membranes could enhance their tumor colonization ability.

Meanwhile, the liver, heart, spleen, lung and kidney tissues were collected and fixed in 4 % paraformaldehyde tissue fixation solution. The histological staining results with hematoxylin and eosin (H&E) were shown in Fig. S6. The results showed no obvious pathological change in each group, indicating that the prepared *EcN*@RBC exhibited excellent biological safety.

### 2.3 Study on the anti-cancer effect of *EcN*@RBC *in vitro*

The RBC membrane not only prolonged the long-term circulation time of *EcN in vivo* but also reduced the inflammatory response and enhanced the colonization ability of bacteria *in vivo*. Building upon these advantages, we further explored the anti-cancer effect of *EcN*@RBC *in vitro*. Colon cancer cell lines are particularly useful for reflecting the carcinogenesis process and the tumor microenvironment within the intestinal environment [59], thereby providing a direct physiological correlation for studying the interactions between intestinal flora and colon cancer. The CT26 cell line is derived from mouse colon cancer and well-established in mouse models [60], which is not only easy to obtain and stable to culture in the laboratory, but also can mimic the characteristics of human colon cancer well and has the convenience of operation. Therefore, CT26 cell line was chosen for both *in vivo* and *in vitro* studies in this experiment. The anti-cancer activity of *EcN*@RBC *in vitro* was evaluated by the CCK8 assay, as shown in Fig. S7. The RBC membrane showed no cytotoxicity against CT26 cells compared with the control group, while the result for *EcN* was similar to that of *EcN*@RBC, resulting in approximately 50 % of cell death. Additionally, we validated the experiment using 4T1 cells and HT29 cells to ensure the reliability of the experimental results. As shown in Figs. S8A and S8C. The results of CCK8 demonstrated significant cytotoxicity of *EcN* and *EcN*@RBC against 4T1 and HT29 cells. Flow cytometry results in Figs. S8B and S8D showed that after treatment with *EcN* and *EcN*@RBC, the apoptosis rates of 4T1 cells were 39.62 % and 30.92 %, respectively. Meanwhile, the apoptosis rates of HT29 cells were 33.4 % and 30.9 %, respectively. These experimental results demonstrated that both *EcN* and *EcN*@RBC have a universal cytotoxic effect on cancer cells.

**Fig. 3:**
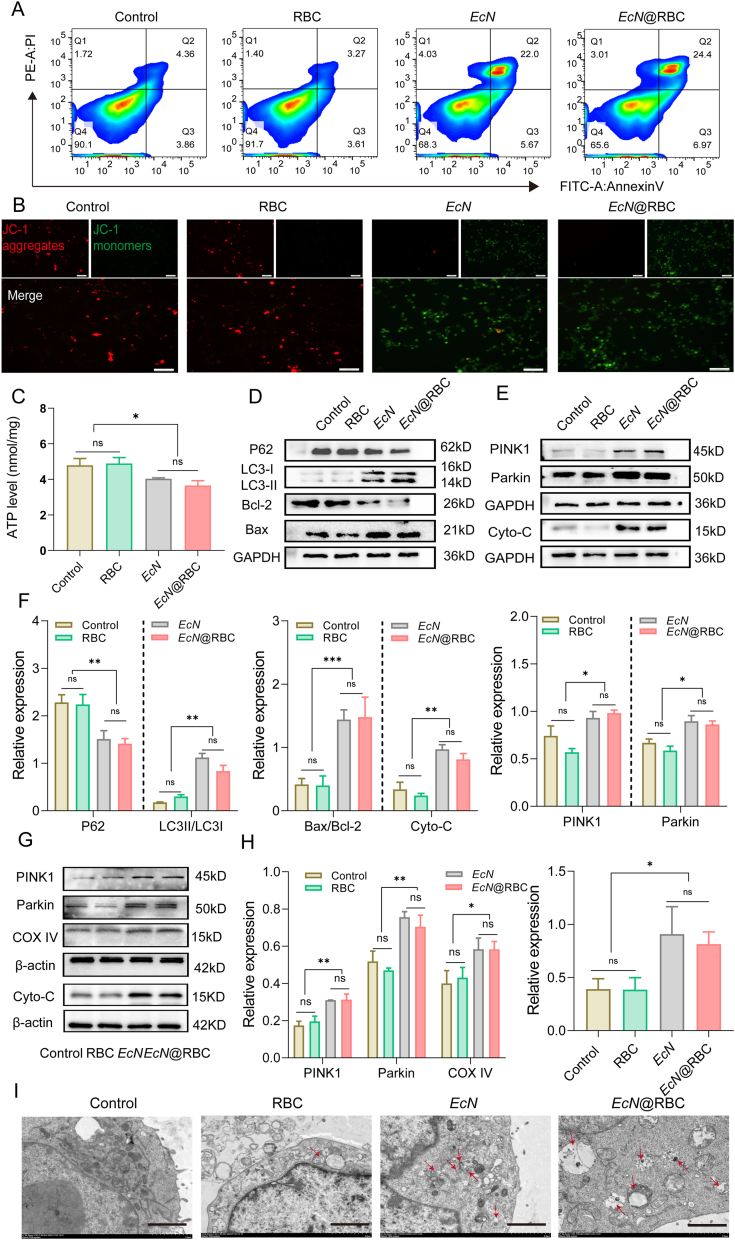
*In vitro* anti-cancer effects of *EcN*@RBC. (A) Flow cytometry analysis of apoptosis in CT26 cells. (B) Assessment of MMP (Scale bar = 50 μm). (C) The release of ATP in different groups of CT26 cell. (D) The expression levels of P62, LC3, Bcl-2, and Bax in different groups. (E) The expression levels of mitophagy proteins PINK1, Parkin, and Cyto- C proteins in different groups. (F) The relative expression level of LC3, P62, Bax/Bcl-2, PINK1, Parkin, and Cyto-C in each group. (G) The expression level of PINK1, Parkin, COX4 and Cyto-C in cell mitochondria in different group. (H) The relative expression level of PINK1, Parkin and COX4, and Cyto-C in cell mitochondria in different group. (I) Representative TEM image of autolysosome. The red arrows represent autolysosomes (Scale bar = 2 μm). Significance was assessed using student's t-test giving p values, ∗p < 0.05, ∗∗p < 0.01, ∗∗∗p < 0.005, ns indicates no statistical significance.

Mitochondrial membrane potential (MMP) serves as a crucial indicator in apoptosis, with its decline typically preceding the appearance of apoptotic features in the cell nucleus [61]. JC-1 is commonly used probe to detect MMP, which exists in two states: monomer and aggregate. The results demonstrated that CT26 cells treated with *EcN* or *EcN*@RBC exhibited strong green fluorescence, indicating a decrease in MMP in the cells (Fig. 3B). MMP is closely related to ATP synthesis [62]. The change of ATP was detected, and the results showed that compared with the control group, there was no significant change in RBC group, while the ATP levels in *EcN* and *EcN*@RBC groups were significantly decreased (Fig. 3C). Mitochondria, known as the powerhouses of the cells, are essential for cells survival [63]. Mitophagy is an essential pathway for controlling mitochondrial quantity and quality, playing a critical role in the survival or death of tumor cells [36]. When mitochondrial are damaged, it can cause a decrease in MMP, resulting in the ubiquitination of mitochondrial outer membrane proteins, thereby mediating the occurrence of mitophagy [64,65]. Here, we primarily explored the relationship between *EcN*@RBC and mitophagy. The classical pathway of mitophagy involves the mediation of PINK1/Parkin [66]. Excessive mitochondrial damage leaded to a decrease of MMP, which stabilized PINK1 in the outer mitochondrial membrane, recruiting the cellular Parkin protein to the mitochondria [67,68]. Therefore, we verified the changes in related mitochondrial protein PINK1/Parkin and autophagy flux of autophagy protein LC3, P62 in cells after treatment. The results revealed increased expression of PINK1/Parkin proteins in *EcN* or *EcN*@RBC group, which are tumor suppressors known to promote cancer cell apoptosis. Additionally, the expression of the autophagy-associated protein LC3II was increased, while P62 expression was decreased (Fig. 3D and E). The relative protein quantification of each group was shown in Fig. 3F. It might be attributed to the activated Parkin protein ubiquitinating various mitochondrial outer membrane proteins, forming a complex with autophagy junction protein P62, facilitated by the LC3 binding, inducing mitophagy [[69], [70], [71]]. Furthermore, the expression of the anti-apoptotic protein Bcl-2 was reduced, while the pro-apoptotic protein Bax was increased. Meanwhile, the expression of caspase-3 and cleaved-caspase-3 in CT26 cells were evaluated. The result in Fig. S9A showed that the level of caspase-3 remained almost unchanged across all groups. Compared with the control and RBC groups, the expression of cleaved-caspase-3 increased in the *EcN* and *EcN*@RBC groups. Surprisingly, the analysis of the semi-quantitative results in Fig. S9B revealed a significant reduction in cleaved-caspase-3 levels in *EcN*-treated cells and *EcN*@RBC-treated cells compared to the control group. This result revealed that *EcN* and *EcN*@RBC activate caspase-3 and induce cleavage, thereby generating active cleaved-caspase-3 to promote apoptosis in cells. Mitochondrial proteins were extracted again to detect the expressions of PINK1 and Parkin in mitochondria. The results were shown in the Fig. 3G. Compared with the control group, the expression levels of PINK1 and Parkin in the RBC group showed no significant difference, whereas a significant upregulation was observed in both the *EcN* and *EcN*@RBC groups.

COX4 (cytochrome oxidase subunit 4) is a subunit of the cytochrome oxidase complex, which is a crucial enzyme in the electron transport chain within mitochondria [72]. Its expression level can be influenced by the status of mitochondrial function [73]. As a component of cytochrome oxidase, COX4, directly affects the efficiency of the electron transport chain and the release of Cyto-C. Moreover, studies have shown that the decrease of MMP can also promote the release of Cyto-C into the cytoplasm, triggering the apoptosis signal cascade [74,75]. Therefore, we assessed the expression of the mitochondrial-specific protein COX4 in cells. As shown in Fig. 3G and H, COX4 expression was elevated in the *EcN* and *EcN*@RBC groups compared to the control and RBC groups. This increased COX4 expression may result from mitochondrial dysfunction, which could affect Cyto-C release. Elevated COX4 levels potentially place tumor cells under greater metabolic stress, thereby enhancing Cyto-C release and promoting apoptosis. In addition, we further explored the protein expression of mitochondrial Cyto-C and the results were consistent with our hypothesis. *EcN* and *EcN*@RBC treatments led to significantly higher levels of Cyto-C compared to the control group (Fig. 3H). This increase may be attributed to enhanced mitophagy and subsequent functional impairment leading to Cyto-C release. Transmission electron microscopy (TEM) analysis revealed an increased number of autophagosomes in the *EcN* and *EcN*@RBC groups compared to the control group (Fig. 3I). Collectively, these findings suggested that *EcN*@RBC may promote cell apoptosis by inducing mitophagy.

### 2.4 Study on the mechanism of *EcN*@RBC regulating mitophagy

**Fig. 4:**
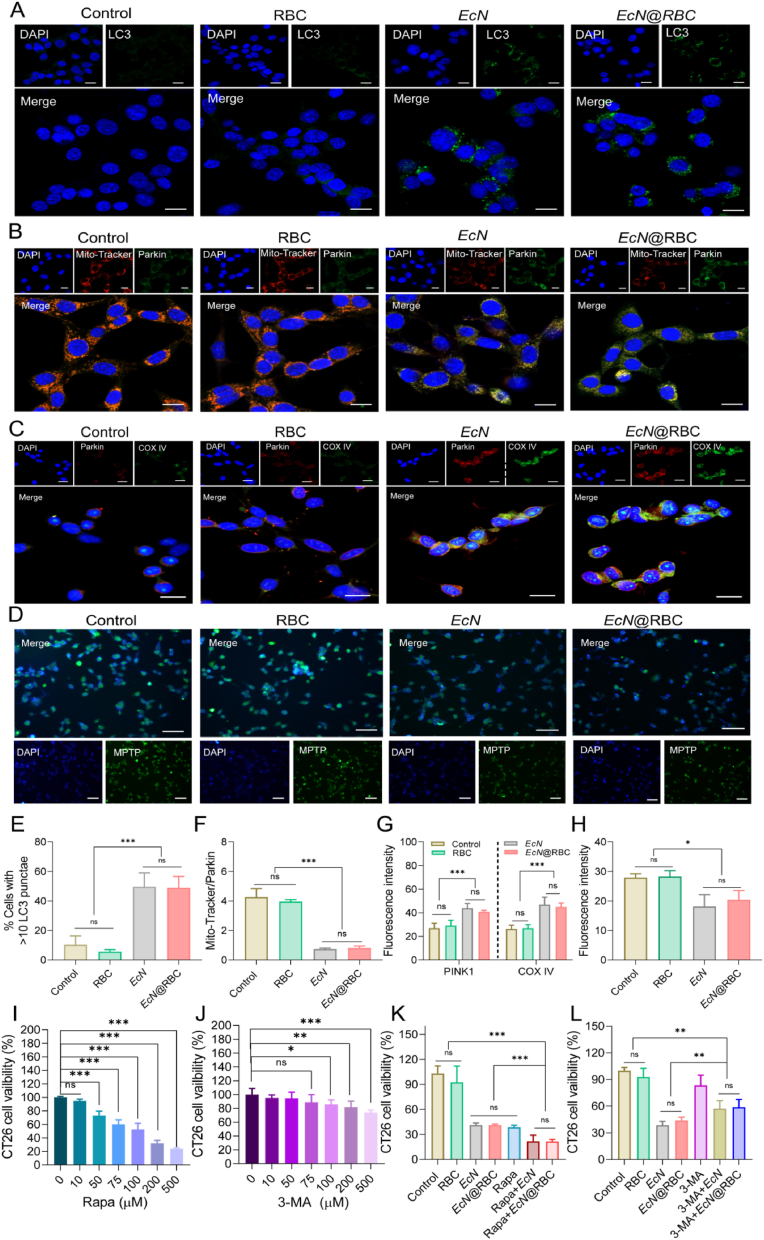
Apoptosis was induced by mitophagy. (A) Immunofluorescence detection of expression of autophagy protein LC3 (green) and nucleus DAPI (blue) (Scale bar = 20 μm). (B) Representative immunofluorescence image of double-labeled mitophagy protein Parkin (green) and mitochondrial marker (Mito-Tracker, red), DAPI (blue, nucleus) were observed by immunofluorescence (Scale bar = 20 μm). (C) Immunofluorescence detection of expression of autophagy protein COX IV (green), Parkin (red) and nucleus DAPI (blue) (Scale bar = 20 μm). (D) MPTP was measured to evaluate mitochondrial permeability. The cytoplasm including mitochondria exhibited strong green fluorescence, and the nucleus exhibits blue fluorescence (Scale bar = 50 μm). (E) LC3 puncta formation was analysed by confocal microscopy. Quantification of LC3 punctae formation in response to the treatments were shown. (F) The ratio of Mito-Tracker/Parkin in different group. (G) The fluorescence intensity of Parkin and COX4 in different group. (H) The fluorescence semi-quantitative of MPTP. (I) Relative cell viability of CT26 cells were treated with various concentrations of the autophagy inhibitor 3-MA. (J) Relative cell viability of CT26 cells were treated with various concentrations of autophagy activator Rapa. (K) Relative cell viability of CT26 cells were treated with the combination of Rapa (50 μm) and *EcN* or *EcN*@RBC. (L) Relative cells viability was treated with the combination of 3-MA (100 μm) and *EcN* or *EcN*@RBC. Significance was assessed using student's t-test, giving p values, ∗p < 0.05, ∗∗p < 0.01, ∗∗∗p < 0.005, ns indicates no statistical significance.

The aforementioned research demonstrated that *EcN* or *EcN*@RBC could induce cellular autophagy. To elucidate the mechanism of the anti-cancer effect of *EcN*@RBC, immunofluorescence was applied to monitor the changes in intracellular LC3. The intensity of green fluorescence was directly proportional to the level of LC3 expression. As shown in Fig. 4A, no significant green fluorescence was detected in the Control and RBC groups, whereas noticeable green fluorescence was observed in the *EcN* and *EcN*@RBC groups. Additionally, the levels of LC3 puncta in CT26 cells was evaluated, as depicted in Fig. 4E, showed a significant increase exclusively in the *EcN* and *EcN*@RBC groups, indicating robust autophagosome formation. In addition, Parkin fluorescence intensity was enhanced after treatment with *EcN*@RBC compared with control, whereas Mito-Tracker staining showed blurred mitochondrial morphology and weak fluorescence intensity in the *EcN* and *EcN*@RBC groups (Fig. 4B and F) and the ratio of Mito-Parkin in the *EcN* and *EcN*@RBC groups was decreased, suggesting that *EcN*@RBC suggesting that *EcN*@RBC may induce mitophagy via Parkin. Meanwhile, the results from Fig. 4C and G indicated increased co-localization of Parkin and COX4 in the *EcN* and *EcN*@RBC groups compared to the control and RBC group. There is also an increase in relative fluorescence intensity, suggesting that Parkin may affect the function of COX4 and other proteins in mitochondria through its ubiquitin ligase activity, thereby indirectly influencing cellular energy metabolism and physiological status in the body. The enhanced expression of COX4 and Parkin in cells signifies activation of mitophagy, indicating their functional interaction in regulating mitochondrial function and metabolism [75]. The decrease in MMP and ATP depletion could both trigger the opening of the mitochondrial outer membrane permeability transition pore (MPTP) [76,77]. MPTP (Mitochondrial Permeability Transition Pore), when opened, increases permeability of the mitochondrial inner membrane. This process is typically associated with mitochondrial dysfunction, such as a sharp decrease in mitochondrial membrane potential or an increase in mitochondrial calcium ion cohesion. This increased permeability lead to the release of Cyto-C from the mitochondria into the cytoplasm, where it binds to apoptotic protease activator 1 (Apaf-1) to form apoptosome [78,79], this complex subsequently activates cysteine-9 of the cysteine family of enzymes, triggering a cascade of cysteine reactions that ultimately induce apoptosis. The previous results suggested that *EcN*@RBC caused mitochondrial damage and excessive mitochondrial consumption, leading to insufficient ATP supply. The degree of mitochondrial MPTP permeability and the expression of Cyto-C was examined. The results showed a decrease in the green fluorescence of MPTP in the *EcN* and *EcN*@RBC group (Fig. 4D and H), along with a significant increase in the expression of Cyto-C (Fig. 3E). These results demonstrated that *EcN* and *EcN*@RBC could induce apoptosis by enhancing mitophagy and causing mitochondrial damage. Based on the aforementioned findings, to further confirm the association between apoptosis and mitophagy induced by *EcN*@RBC, we selected the autophagy inhibitor 3-methyladenine (3-MA) and the autophagy promoter rapamycin (Rapamycin, Rapa) to validate their effects on cells. The results exhibited that Rapa group had a dose-dependent effect on the proliferation of CT26 cells, while 3-MA group had no obvious inhibitory effect on CT26 cells (Fig. 4I and J). To further validate the efficacy of the combination therapy in *vitro*, the Rapa or 3-MA were applied in combination with *EcN* or *EcN*@RBC to CT26 cells. The results demonstrated that the combination of Rapa *EcN*@RBC had a pronounced effect than the combination of 3-MA and *EcN*@RBC (Fig. 4K and L).

Moreover, we utilized 3-MA and Rapa to assess the potential relationship between apoptosis and mitophagy by western blot. The results are shown in Fig. S10. After treating CT26 cells with *EcN* and *EcN*@RBC, the levels of mitophagy protein PINK1/Parkin and autophagosome marker protein LC3 were increased, and the increases of these proteins were more obvious after the addition of Rapa. Conversely, when 3-MA was added to the treatments with *EcN* and *EcN*@RBC, the expression of these proteins was reversed. At the same time, treatment with *EcN* and *EcN*@RBC also led to an increase in the pro-apoptotic protein Bax and a decrease in the anti-apoptotic protein Bcl-2, with the Bax/Bcl-2 ratio showing a more significant increase upon the addition of Rapa. Furthermore, the addition of 3-MA to the treatments with *EcN* and *EcN*@RBC reversed the Bax/Bcl-2 ratio. Z-VAD-FMK is a widely used cysteine protease inhibitor in cell biology research, commonly employed to study the apoptotic process by inhibiting the activation of caspase3 and thereby halting apoptosis progression [80]. We utilized the pan-caspase inhibitor Z-VAD-FMK to explore the relationship between autophagy and apoptosis. The result in Fig. S11A was depicted that compared to the PBS group, the caspase inhibitor Z-VAD-FMK had no effect on the changes in LC3 and P62, whereas there was a decrease in P62 protein expression and an increase in LC3 protein expression in the *EcN* and *EcN*@RBC groups. With or without Z-VAD-FMK did not affect the expression of LC3 and P62 in *EcN* and *EcN*@RBC, and the relative protein expression was shown in Fig. S11C. These findings indicated that inhibiting apoptosis may not influence the changes of autophagy. Furthermore, we also examined the expression of caspase-3 and cleaved-caspase-3 in each group, as described in Fig. S11B. Compared with the PBS and RBC groups, the expression of cleaved caspase-3 decreased after the addition of Z-VAD-FMK in the *EcN* and *EcN*@RBC groups (Fig. S11D), clearly demonstrating that Z-VAD-FMK effectively inhibits apoptosis. Autophagy and apoptosis are indeed interconnected in cell biology. When cells are exposed to prolonged stress or damage, autophagy can influence cell survival duration, thereby indirectly impacting the occurrence of apoptosis [81]. Our experimental results also suggested that Z-VAD-FMK inhibiting apoptosis does not affect cellular autophagy, whereas 3-MA inhibiting autophagy affect cellular apoptosis. These results indicated that *EcN* and *EcN*@RBC induce apoptosis in CT26 cells by enhancing mitophagy. MMP result demonstrated that *EcN*@RBC synergistically promoted cell apoptosis when combined with Rapa (Fig. S12). In brief, the above results suggested that *EcN*@RBC could induce the entry of PINK1/Parkin into cells by reducing MMP and releasing ATP, thereby increasing the development of MPTP and releasing Cyto-C to promote apoptosis of colon cancer cells.

### 2.5 Study on the anti-tumor effect of *EcN*@RBC *in vivo*

**Fig. 5:**
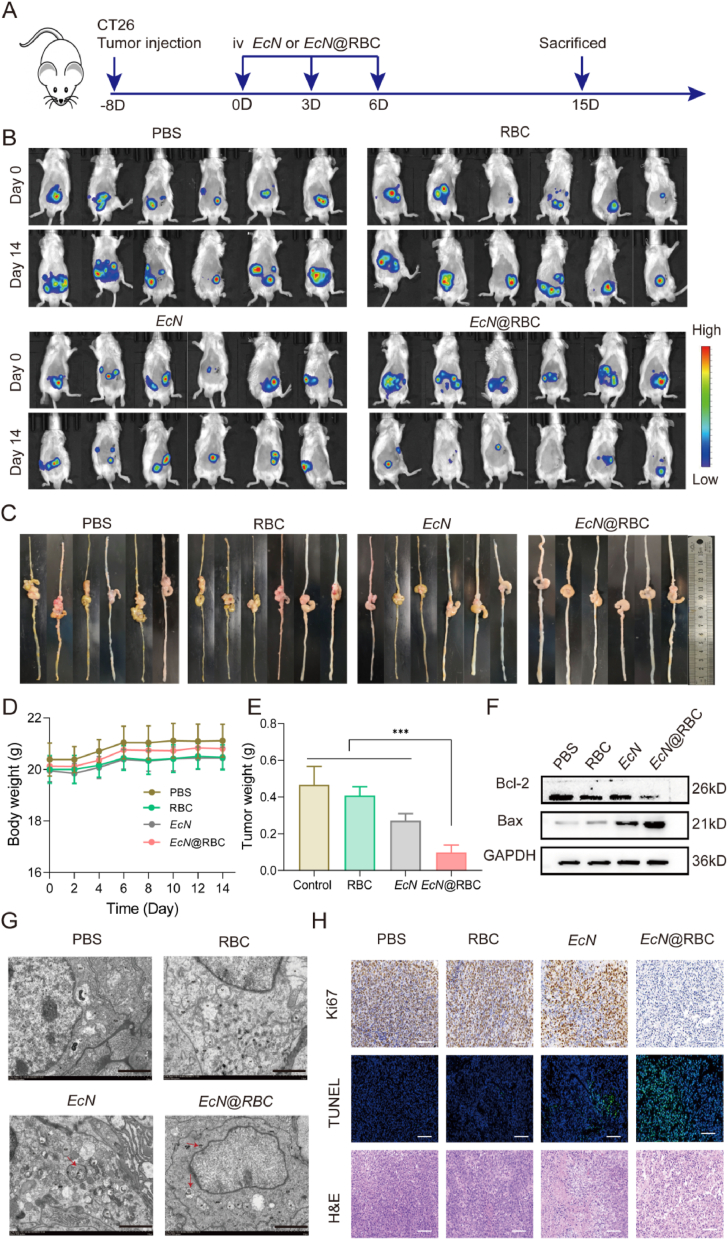
Antit-umor effect of *EcN*@RBC *in vivo*. (A) Schematic diagram of experimental design. (B) Images of mice IVIS *in vivo*. (C) The photograph of tumor tissue. (D) The body weight of mice during treatment. (E) Tumor weight in mice. (F) The changes of apoptosis proteins Bcl-2 and Bax in tumor. (G) Representative TEM image of autolysosome. The red arrows represent autolysosomes (Scale bar = 2 μm). (H) Immunohistochemical staining of Ki6, TUNEL, and H&E staining in tumor tissues (Scale bar = 20 μm). Significance was assessed using student's t-test, giving p values, ∗p < 0.05, ∗∗p < 0.01, ∗∗∗p < 0.005.

In light of the demonstrated excellent tumor colonization ability and anti-cancer effect of *EcN*@RBC *in vitro*, further evaluation of its anti-tumor efficacy *in vivo* was conducted. Tumor-bearing mice were randomly assigned to four groups: PBS, RBC, *EcN*, and *EcN*@RBC. The experimental process was depicted in Fig. 5A. On the 8th day after tumor formation, mice were intravenously injected with the corresponding drug every three days for a total of three times, and tumor changes were observed using *in vivo* imaging system (Fig. 5B). After 15 days, the mice were euthanized, and the tumor tissues were collected, photographed, and weighed. The results showed a significant reduction in tumor size in the *EcN*@RBC group (Fig. 5C and E). Consistent with the observations from the *in vivo* imaging system in Fig. 5B, which depicted rapid tumor growth in the PBS and RBC groups but significant tumor growth inhibition in the *EcN*@RBC group, suggesting enhanced anti-tumor efficacy due to the high concentration of *EcN* at the tumor site. During the administration period, the body weight of mice was measured every other day. The result showed that there were no significant changes in the body weight of the mice in each group, indicating no serious systemic toxicity of *EcN*@RBC (Fig. 5D). More convincingly, as shown in Fig. 5F, we detected the changes of pro-apoptotic protein Bax and anti-apoptotic protein Bcl-2 in tumor tissues. The Bax/Bcl-2 ratio was elevated in the *EcN* and *EcN*@RBC groups compared with the PBS and RBC groups. Moreover, the Bax/Bcl-2 ratio was notably higher in the *EcN*@RBC group compared to the *EcN* group (Fig. S13A). An increased Bax/Bcl-2 ratio can lead to changes in mitochondrial membrane permeability [82,83], the release of apoptotic mediators, and ultimately the induction of apoptosis, indicating a significant anti-tumor effect of *EcN*@RBC. Consequently, we examined changes in autophagosomes across the tumor groups. TEM analysis revealed a significant increase in the number of autolysosomes in the *EcN*@RBC group, accompanied by notable alterations in mitochondrial morphology (Fig. 5G). Subsequently, the expression of relevant autophagy proteins and apoptosis proteins were evaluated. The results demonstrated a significant upregulation of PINK1/Parkin and LC3, while P62 was significantly downregulated in the tumor tissues of the *EcN*@RBC group compared with other groups (Fig. S13B). The correlation quantification of each protein in each group was shown in Fig. S13C. Immunofluorescence of tissues analysis provided a visual representation of the fluorescence expression of key autophagy proteins Parkin and LC3 in tumor tissues of the *EcN*@RBC group consistent with the protein quantitative analysis results (Fig. S14). These results illuminated that *EcN*@RBC could exerted antitumor effects by enhancing mitophagy. Additionally, the tumor morphology and cell death were evaluated by H&E and TUNEL staining after treatment, as shown in Fig. 5H. Minimal cell death was observed in the PBS group, while the *EcN +* RBC groups exhibited partial cell death. Notably, the *EcN*@RBC group displayed loose cellular arrangement, with evident nuclear consolidation. What's more, the TUNEL staining also presented extensive necrosis in the *EcN*@RBC group, suggesting that the *EcN*@RBC group could effectively treat colon cancer. Ki67, primarily assessing tumor cell proliferation, showed reduced staining intensity in the *EcN*@RBC group. The results indicated that *EcN*@RBC may induce tumor cells apoptosis through mediating mitophagy, and the *EcN*@RBC possessed significant anti-tumor potential.

### 2.6 Study on anti-tumor of the autophagy modulators combiantion with *EcN*@RBC *in vivo*

**Fig. 6:**
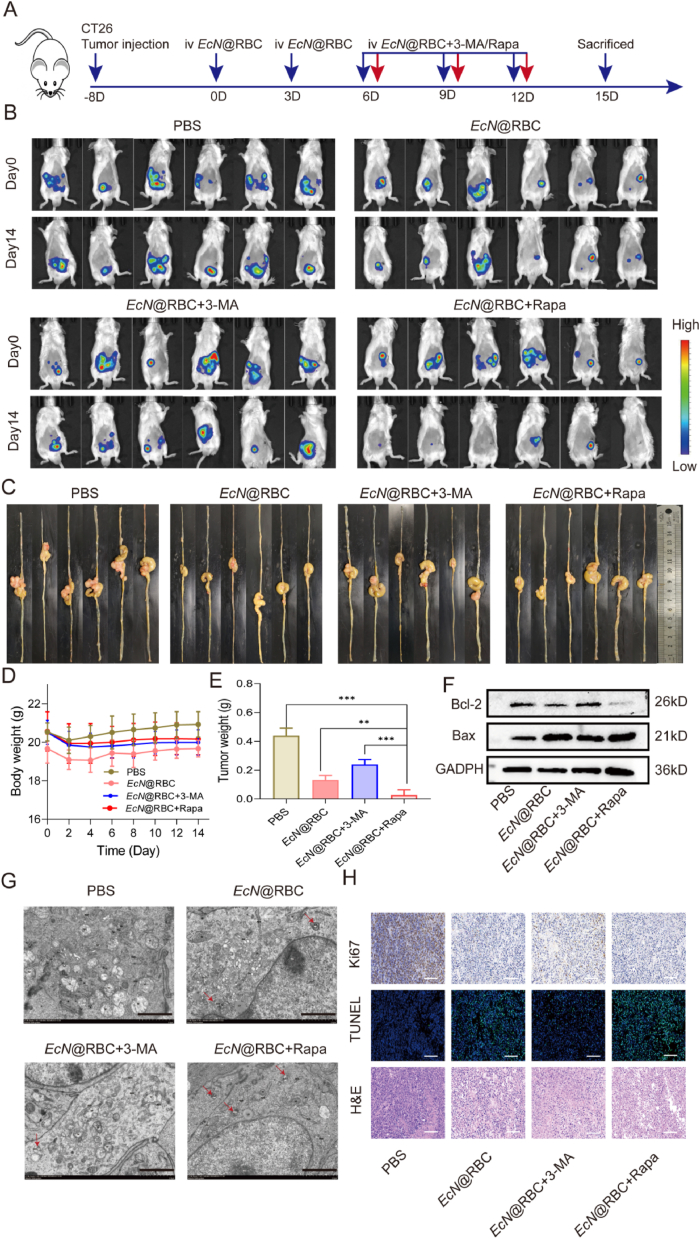
Anti-tumor mechanism of *EcN*@RBC *in vivo*. (A) Schematic diagram of experimental design. (B) Images of mice IVIS *in vivo*. (C) Photos of tumor tissue. (D) The body weight of mice during treatment. (E) Tumor weight of the mice. (F) The changes of Bcl-2 and Bax in tumor. (G) Representative TEM image of autolysosome. The red arrows represent autolysosomes (Scale bar = 2 μm). (H) Ki67, TUNEL and HE staining of tumor tissue (Scale bar = 20 μm). Significance was assessed using student's t-test, giving p values, ∗p < 0.05, ∗∗p < 0.01, ∗∗∗p < 0.005.

The above results suggested that *EcN*@RBC might impede tumor development by mediating mitophagy. To investigated whether *EcN*@RBC promotes tumor cells apoptosis through the mitophagy pathway, we explored the anti-tumor efficacy of autophagy regulators combined with *EcN*@RBC *in vivo*. Tumor-bearing mice were randomly assigned into four groups as depicted in Fig. 6A. On days 0, 3, 6, 9, and 12, the animals were injected intravenously with *EcN*@RBC. On days 6, 9, and 12, mice received intraperitoneal injections of Rapa or 3-MA respectively, while being kept on a normal diet. The tumor changes were observed *in vivo* by IVIS after the completion of the treatment (Fig. 6B). After 15 days, mice from each group were euthanized, and tumor tissues were collected, photographed, and weighed. The results showed the tumor growth was rapid in the PBS group, while significantly inhibited was observed in the other groups. However, the tumor growth rate of *EcN*@RBC in combined with 3-MA was faster compared to that of *EcN*@RBC group. Notably, while the tumor growth was significantly inhibited in the group receiving the combination of *EcN*@RBC and Rapa, indicating that *EcN*@RBC affects tumor growth through autophagy (Fig. 6C and E). These findings were consistent with those presented in Fig. 6B, demonstrating a substantial reduction in tumor size in the group treated with *EcN*@RBC combined with Rapa group. Additionally, there was no significant decrease in body weight among the groups during treatment (Fig. 6D).

Meanwhile, the changes of autolysosomes were observed by TEM in Fig. 6G. The results indicated an increased level of autolysosomes in all treatment groups except the control group, with the most significant effects observed in the group treated with *EcN*@RBC combined with Rapa, indicating severe mitochondrial damage. Moreover, protein samples extracted from the tumor tissues were subjected to western blotting analysis to examine the expression of autophagy-related proteins and apoptosis proteins. The results indicated that in comparison to the *EcN*@RBC group, the treatment combining *EcN*@RBC with Rapa led to an upregulation of PINK1/Parkin and LC3 expression, and a downregulation of P62 expression. Conversely, the administration of 3-MA inhibited the autophagy induced by *EcN*@RBC and contributed to the promotion of tumor growth (Fig. S15B). The relative quantification of proteins in each group was referred to in Fig. S15C. Immunofluorescence of tissues analysis provided an intuitive representation of key autophagy proteins Parkin and LC3 in tumor tissues of the group treated with *EcN*@RBC combined with Rapa, consistent with the protein quantitative analysis results (Fig. S16). More surprising, as shown in Fig. 6F, relevant apoptotic proteins in each group of tumor tissues were examined. Compared to the PBS group, the Bax/Bcl-2 ratio decreased in the *EcN*@RBC group, while the ratio was highest in the *EcN*@RBC and Rapa combination group. Conversely, 3-MA reversed the anti-tumor effect of *EcN*@RBC (Fig. S15A). These results suggested that inhibiting autophagy can attenuate the anti-tumor effect of *EcN*@RBC, whereas enhancing autophagy can strengthen its anti-tumor efficacy. Furthermore, the tumor morphology and cell death were evaluated by H&E and TUNEL staining after treatment, as shown in Fig. 6H. The results demonstrated reduced proliferation and increased apoptosis in the tumor tissue of the group treated with *EcN*@RBC combined with Rapa compared with other groups, indicating that the combination of *EcN*@RBC with Rapa induced more powerful antitumor efficacy. In summary, these results suggested that *EcN*@RBC possesseed an antitumor effect by enhancing mitophagy, which was significantly enhanced when combined with Rapa, synergistically inducing mitophagy and tumor apoptosis.

## 3 Conclusion

In this study, we described a Trojan horse *EcN*@RBC prepared by encapsulating *EcN* probiotic with RBC for the treatment of colon cancer. The Trojan horse-like camouflage of *EcN*@RBC not only mitigates the systemic inflammatory response but also extends its circulation in the bloodstream, facilitating better colonization of anaerobic bacteria in the tumor microenvironment. Additionally, we discovered that *EcN*@RBC could induced increased autolysosomes, mitochondrial swelling, and vacuolization in CT26 cells, it was also found that *EcN*@RBC caused the increase of mitochondrial protein PINK1/Parkin. Interestingly, the PINK1/Parkin-mediated mitophagy pathway activated by mitochondrial depolarization and resulted in ubiquitination of various outer mitochondrial membrane proteins. The activated Parkin bound P62 and LC3, inducing mitophagy and subsequent cell apoptosis. Furthermore, both *in vivo* and *in vitro* experiments demonstrated that *EcN*@RBC upregulated mitophagy through PINK1/Parkin induction, leading to cell apoptosis. However, further research is warranted to elucidate the specific component of *EcN*@RBC that plays a significant role in its anti-tumor effects.

## 4 Materials and methods

### 4.1 Cell, strains and animals

CT26 cells were provided by the School of Pharmacy, Fudan University, and cultured in 1640 medium (the culture organisms) which were fed with 10 % fetal bovine serum (FBS) and 1 % penicillin streptomycin. Cells were cultured in a moist environment of 37 °C with 5 % CO_2._

*EcN* strain was purchased from Beijing Biaowei Biotechnology Co, LTD. The *EcN* strain was cultured in LB medium at 37 °C overnight, then diluted 1:100 into fresh LB medium for 3 h. *EcN* were centrifuged at 5000 g for 10 min and resuspended in pre-cooled PBS. Bacterial counts were determined by diluting bacterial suspensions, incubated them on LB agar plates overnight and calculating CFU.

Balb/c mice (male, 6–8 weeks of age) were purchased from Shanghai Jesijie Laboratory Animal Co, LTD. The mice were raised in a sterile environment with a light and dark cycle of 12 h and free access to food and water. All animal experiments were reviewed and approved by the Fudan University Laboratory Animal Ethics Committee.

### 4.2 Preparation of EcN@RBC

The extraction of the erythrocyte membrane according to the reported method is straightforward with some modifications [48]. Whole blood was collected from the retroorbital cluster of Balb/c mice and diluted in precooled phosphate saline buffer containing heparin, then it was centrifuged at 800 g for 5 min to remove the supernatant and collect red blood cells, repeating 3 times. The solution was rinsed with cold PBS containing 1 mM EDTA·2Na and centrifuged at 2500 g for 3 times for 5 min each time. The obtained RBC was dispersed into isotonic solution, then added with 4-fold the volume of hypotonic solution, rotated and centrifuged for 10 min at 12000 g, and the RBC membrane was obtained by repeated centrifugation to remove hemoglobin. The above experimental operations were carried out at 4 °C and stored at −20 °C for reserve.

*EcN* was diluted to OD of about 0.15 after 3 h of subculture at 37 °C, the above bacterial solution (1 mL) was washed twice with pre-cooled PBS, resuspended to 1 mL, and mixed with 1 mL erythrocyte membrane prepared from Balb/c mouse blood. The mixed solution was extruded 20 times with polycarbonate porous membrane (pore size was 1 μm). The extruded bare *EcN* serves as the control.

### 4.3 The characterization of EcN@RBC

The particle size and ζ potential of *EcN* and *EcN*@RBC were measured by DLS (Malvern Zetasizer Nano ZS90). *EcN* and *EcN*@RBC were characterized by TEM (FEI Tecnai 12) and SEM (ZEISS GeminiSEM 300). Detailed TEM and SEM samples were prepared as follows, first, *EcN* and *EcN*@RBC were fixed with PBS containing 4 % glutaraldehyde at 4 °C. The 50 μL fixed sample was placed on the silicon wafer, rinsed with ultra-pure water and dehydrated in the ethanol/aqueous solution. The ethanol content increased from 30 % to 100 % tert-butanol (TBA)/aqueous solution, and the TBA content increased from 40 % to 100 %. Finally, TBA was removed from the sample by freeze-drying. In flow cytometry. *EcN*@RBC was resuspended in PBS and incubated with FITC-anti-CD47-antibody (127503, Biolegend) at 4 °C for 30 min. Rinsed with PBS prior to examination.

### 4.4 Detection of live and dead bacteria

*EcN, EcN*@RBC (*EcN*, 1 × 10^6^ CFU/mL). Were incubated in a constant temperature shaking bed at 37 °C for 24 h to reach the plateau stage, centrifuged at 5000 g for 10 min, the bacterial solution was re-suspended in sodium chloride solution and centrifuged three times, for the last time, the solution was suspended in 1 mL sodium chloride solution, appropriate amount of bacterial solution was taken and mixed with 5 μL SY709 and 5 μL PI solution, incubated at room temperature for 15 min away from light, appropriate amount of bacterial solution was taken on the slide, the slide was placed on the cover, and observed under LSCM.

### 4.5 Western blotting

Briefly, the proteins of all samples were cleaved on ice for 15 min using protein RIPA cleavage buffer (Beyotime P0013B) and then centrifuged at 12000 g for more than 10 min. The protein concentrations of the samples were determined using the BCA protein Quantification kit (Beyotime P0009). All samples were mixed with loading buffer and run on sodium dodecyl sulfate polyacrylamide (SDS-PAGE) gel electrophoresis. The isolated samples were transferred to a polyvinylidene difluoride (PVDF) membrane by blocking the PVDF membrane with 5 % skim milk at room temperature for 1 h and then detected with the relevant primary antibody: CD47 (Abcam, ab175388), P62 (Cell Signaling Technology, 23214S), LC3 (Cell Signaling Technology, 3868S), PINK1 (Proteintech, 23274-1-AP), Parkin (Proteintech, 14060-1-AP), Bax (Cell Signaling Technology, 14796S), Bcl-2 (Cell Signaling Technology, 15071S), Cytochrome C (Cell Signaling Technology, 11940S), then washed three times on PBS the next day, and exposed after the secondary antibody was incubated.

### 4.6 Growth curves of EcN@RBC

Bacteria were collected, cleaned with cold PBS, and then mixed with pre-prepared erythrocyte membranes, as described in preparation *EcN*, *EcN* + RBC and *EcN*@RBC were both diluted in LB, incubated at 37 °C, and gently shaken. The growth curves were drawn through recording the OD value of 600 nm at various time points by a multifunctional microplate reader.

### 4.7 Growth viability assay of EcN@RBC

Before colony counting *EcN,EcN* + RBC and *EcN*@RBC were applied to LB agar plates and incubated at 37 °C overnight. For cell activity determination, 100 μL of *EcN*, *EcN* + RBC and *EcN*@RBC (*EcN*, 1 × 10^6^ CFU/mL) were inoculated on 96-well plate and cultured at 37 °C. The 10 μL AlamarBlue solution was added to each well. Meanwhile, od values at different time points were detected by multifunctional enzyme marker (ex = 560 nm, em = 590 nm).

### 4.8 Hematological testing

Including routine blood test and serum biochemical analysis. To ensure the safety of *EcN*@RBC, PBS, RBC, *EcN*, *EcN* + RBC and *EcN*@RBC were injected intravenously into CT26 carcinoma in orthotopic mice (*EcN*, 1 × 10^6^ CFU/mL). Then 50 μL of blood was collected from the orbit of each mouse within the specified time, and the AST, ALT and ALP of erythrocyte, leukocyte, platelet were determined by standard animal hematology analyzer.

### 4.9 Elisa assay

In order to measure the inflammatory response caused by *EcN*@RBC, 100 μL of blood was extracted from Balb/c mice at a predetermined time, incubated at 37 °C for 30 min, centrifuged at 3000 g for 5 min, and then treated with a commercially available ELISA kit (MultiSciences Biotech, China) to detect IL-6, IL-10, TNF-*α*, IFN-γ and IL-1β.

### 4.10 Blood reservation assay

To assess the blood retention capacity of *EcN*@RBC. Balb/c male mice aged 6–8 weeks were injected intravenously with PBS, RBC, *EcN*, *EcN* + RBC and *EcN*@RBC (*EcN*, 100 μL, 1 × 10^6^ CFU/mL). 50 μL of orbital blood was collected at different time points (0 h, 1 h, 4 h, 12 h, 48 h, and 72 h), 10 μL of blood was collected from each mouse and diluted with PBS and 50 μL of blood was coated on LB agar plate, 37 °C incubated overnight, and bacteria were counted the next day.

### 4.11 Biological distribution study

To investigate the biological distribution of *EcN* and *EcN*@RBC in organisms, Mice with CT26 in orthotopic colon tumor model were injected intravenously with PBS, RBC, *EcN*, *EcN* + RBC and *EcN*@RBC (*EcN*, 100 μL, 1 × 10^6^ CFU/mL). Five mice in each group were sacrificed at a predetermined time point (0 h, 2 h, 6 h, 24 h, 48 h, 72 h, and 120 h), and liver, heart, spleen, lung, kidney and tumor tissues were collected. In addition, 10 μL homogenate was taken for continuous dilution with PBS and 50 μL of the diluted homogenates were coated on LB agar plate. Incubated at 37 °C overnight and count bacteria the next day.

### 4.12 Hematoxylin eeosin assay

Hematoxylin eosin (H&E) was used to analyze tumor aggregation and distribution, the liver, heart, spleen, lung and kidney were stained HE, then scaned imaging to observe histopathologic changes.

### 4.13 CCK8 assay

100 μL 5000 cells were added to each well of the six-well plate. According to the needs of the experiment, specific drugs were added to each well of the six-well plate for a certain period of time, and 10 μL CCK8 solution was added to each well. The cells were incubated for 1–2 h and absorbance was measured at 450 nm. *In vitro* experiments are usually conducted in a sterile environment to avoid microbial contamination. Before adding probiotics, all the culture media, cell cultures, and small containers need to undergo strictly disinfection and sterilization procedures. Furthermore, the experimenter should also follow the aseptic procedures, such as wearing sterile gloves and using sterile tools. Secondly, regarding the treatment of probiotics and tumor cells, probiotics are usually added to cell cultures in the form of suspensions.

### 4.14 Immunofluorescence

Immunofluorescence staining was performed by placing CT26 cells onto a six-well plate. The treatment was performed as described above. In order to label mitochondria. Mito-Tracker Red CMXRos (Beyotime C1035) was added to CT26 cells and incubated for 30 min, and sequentially fixed with 4 % tissue fixator for 10min, tronatone was permeated by 0.1 % for 30 min, then blocked by 5 % BSA for 1 h, incubated with anti-LC3 antibody (1:100) anti-Parkin (1:100) at 4 °C overnight, incubated with fitc-goat anti-rabbit antibody for 1 h, stained with DAPI for 10 min, and observed under fluorescence microscope.

### 4.15 Flow cytometry

Flow detection was performed using Biyuntian kit (C1062S), 6-well plate cells were collected in a centrifuge tube, centrifuged 1000*g* for 5 min, the supernatant was abandoned, and the cells were suspended with 195 μL Annexin V-FITC binding solution and mixed with 5 μL Annexin V-FITC, then added with 5 μL Annexin V-FITC, incubated at room temperature (20–25 °C) away from light for 10–20 min, and test with flow cytometry immediately.

### 4.16 ATP assay

The culture solution was removed, and the lysate was added at a ratio of 200 μL per well of the 6-well plate to lysate the cells. After cracking, centrifuged at 4 °C 12000 g for 5 min, take the supernatant, melt the reagent to be used on the ice bath, and dilute the ATP standard solution into an appropriate concentration gradient with ATP detection cracking solution. Calculated the ATP concentration of the sample according to the instructions of Beyotime S0026.

### 4.17 MPTP assay

Firstly, the CT26 cells were inoculated in six-well plates (10^5^ cells/well) and divided into 4 groups: Control, RBC, *EcN*, and *EcN*@RBC (*EcN*, 1 × 10^6^ CFU/mL), each group was incubated at 37 °C, 5 % CO2 for 6 h and then aspirated off the culture solution, and the cells were washed with PBS for 1–2 times. Subsequently, 1 mL of the configured Calcein AM staining solution was added, and the cells were gently shaken to make the dye cover all the cells uniformly, and incubated for 30 min at 37 °C in the dark. After incubation, the medium was replaced with fresh, pre-warmed medium at 37 °C, and the cells were incubated again in the dark at 37 °C for 30 min to allow intracellular esterases to fully hydrolyze Calcein AM, producing green fluorescent Calcein. The culture medium was removed and washed with PBS for 2–3 times, and then the detection buffer was added to be observed under a fluorescence microscope.

### 4.18 Establishment of an orthotopic colon cancer model in mice

Whole cell intracytoplasmic injection. The mice were anesthetized with isoflurane and made an incision in the left lower abdomen. The incision was disinfected, and the cecum was found to have been removed from the left lower abdomen and dragged out of the incision. 100 μL of 5 × 10^6^ CT26 cells suspended in normal saline were injected into the subserous membrane of the blood-rich region in the middle of the cecum using a 1 mL syringe. After the needle was pulled out after injection, a small cotton ball was used to press for 30 s. After the injection liquid was absorbed by the intestinal wall, the abdominal cavity was cleaned with normal saline and the cecum was reset to avoid pressure and close the abdominal cavity. Mice in the sham surgery group underwent the same surgical procedure except for the cell injection. The whole procedure followed the principles of aseptic surgery and no antibiotics were used after the operation. After the modeling, all mice were placed in the SPF animal house for routine feeding for 8 days, and tumor growth was observed by IVIS *in vivo*.

### 4.19 Histological analysis

The tissues of mice in each group were collected, fixed with 4 % paraformaldehyde for more than 48 h, paraffin embedded sections were stained by H&E and Tunel, and the paraffin embedded sections were stained by immunofluorescence such as dewaxing, penetration and blocking. Subsequently, they were stained with DAPI, Parkin and LC3, observed and photographed with Nikon inverted fluorescence microscope (Tokyo, Japan).

### 4.20 Anti-cancer study *in vitro*

The *in vitro* anti-cancer activity of *EcN*@RBC was evaluated by CCK8. The expression of autolysosome and mitochondrial change were observed by TEM, mitochondrial membrane potential staining, and apoptosis was detected by flow cytometry. CT26 cells were placed on a six-well plate and incubated with *EcN* or *EcN*@RBC for 6 h, which was washed twice with pre-cooled PBS, cleaved with rapi lysate for 30 min, and centrifuged at 12000 g for 10 min. Protein concentration was determined by BCA (Beyotime P0010S), then western blotting was used to detect the expression of related proteins PINK1, Parkin, LC3, P62, Cytochrome C, Bax and Bcl-2 in CT26.

### 4.21 Establishment of tumor model and evaluation of therapeutic effect

The antitumor properties of *EcN*@RBC were evaluated in CT26 mice with in orthotopic colon cancer. In our experiment, the mice were anesthetized with isoflurane and was made in the lower left abdomen. 100 μL of 5 × 10^6^ colon cancer cells suspended in normal saline were injected into the subserous membrane of the blood-rich region in the middle of the cecum using a 1 mL syringe. After the needle was withdrawn from the injection, a small cotton ball was used to press for 30 s. After the injection liquid was absorbed by the intestinal wall, the abdominal cavity was cleaned with normal saline and the cecum was reset to avoid pressure and close the abdominal cavity. Mice with carcinoma in orthotopic were randomized at a predetermined time to observe tumor changes by IVIS *in vivo* (n = 6). Then the mice were injected intravenously (*EcN*, 1 × 10^6^ CFU/mL) with PBS, RBC, *EcN* or *EcN*@RBC 100 μL every 3 days, and their weight was measured three times every 2 days. After 15 days, the mice were euthanized, the intestinal tissue was removed, and the tumors were photographed and weighed. Western blotting was used to detect the expression of relavant proteins in the tumor tissue of each group, and Ki67 was used to detect the proliferation of each group.

### 4.22 Study on apoptosis induced by mitophagy

CT26 mice with carcinoma in orthotopic were divided into four groups (PBS, *EcN*, *EcN*+3-MA, *EcN* + Rapa) to observe the tumor changes at a predetermined time. PBS, *EcN* and *EcN*@RBC (100 μL, 1 × 10^6^ CFU/mL/piece) were injected intravenously on days 0, 3, and 6, and 3-MA (MCE HY-19312, 15 mg/kg/piece) or Rapa (MCE HY-10219, 5 mg/kg/piece) were injected intritoneally on days 6, 9, and 12 respectively. On day 15, the mice were euthanized, and in orthotopic colon tissue was collected, and the tumor was stripped for photo weighing. Western blotting and immunofluorescence were used to detect the expression level of LC3 or Parkin protein in the tumor tissue to reflect the degree of mitophagy. HE and proliferating cell nuclear antigen (Ki67) for tumor staining.

### 4.23 Statistical analysis

All statistical analyses were performed using the GraphPad Prism 8 software package. All error bars used in this study are means ± SD of at least three independent experiments. Statistically significant P values are indicated in the figures and/or legends as ∗p < 0.05, ∗∗p < 0.01 and ∗∗∗p < 0.005, ns indicates no statistical significance.

## Funding

This work was financially supported by the 10.13039/100014717National Natural Science Foundation of China (32172894), Academic Leaders Training Program of Pudong Health Committee of Shanghai(Grant No. PWRd2022-09, the Scientific Research Project of Shanghai Pudong Hospital (YJYJRC202106), and Key Discipline Construction Project of Pudong Health Bureau of Shanghai: Clinical Pharmacy (Grant No.PWZxk2022-27), and Clinical Pharmacy Key Specialized subject Construction Project of Pudong Hospital affiliated to Fudan University (Grant No. Tszk2020-05)

## CRediT authorship contribution statement

**Yang Wang:** Writing – review & editing, Writing – original draft, Methodology, Data curation. **Yao Liu:** Writing – review & editing, Validation. **Xiaomin Su:** Writing – review & editing, Resources. **Lili Niu:** Methodology. **Nannan Li:** Data curation. **Ce Xu:** Resources. **Zanya Sun:** Software. **Huishu Guo:** Validation. **Shun Shen:** Validation. **Minghua Yu:** Investigation.

## Declaration of competing interest

No potential conflict of interest was reported by the authors.

## Data Availability

The authors do not have permission to share data.
