# Supplementary material for: Non-pathogenic Trojan horse Nissle1917 triggers mitophagy through PINK1/Parkin pathway to discourage colon cancer
Source: Mater Today Bio. 2024 Sep 27;29:101273. doi: 10.1016/j.mtbio.2024.101273 (PMC11480251; doi:10.1016/j.mtbio.2024.101273)
Supplement: Multimedia component 1 [file mmc1.docx]

Supporting Information of

**Non-pathogenic Trojan horse *Nissle1917* triggers mitophagy through PINK1/Parkin pathway to discourage colon cancer**

Yang Wang ^a,b,^^1^, Yao Liu ^c,^^1^, Xiaomin Su ^a,b,1^, Lili Niu ^a,b^, Nannan Li ^a,b^, Ce Xu ^a,b^, Zanya Sun ^a,b^, Huishu Guo ^b^*, Shun Shen ^a^*, Minghua Yu ^d^*

^a.^ Pharmacy Department, Shanghai Pudong Hospital, Fudan University Pudong Medical Center, Shanghai, 201399, China

^b.^ Central Laboratory, First Affiliated Hospital, Institute (college) of Integrative Medicine, Dalian Medical University, Dalian 116011, China

^c.^ Clinical Oncology Center, Shanghai Municipal Hospital of TCM, Shanghai University of Traditional Chinese Medicine, Shanghai 200071, China

^d.^ Fudan University Clinical Research Center for Cell-based Immunotherapy & Department of Oncology, Fudan University Pudong Medical Center, Shanghai 201399, PR. China

^b*^ Email: guohuishu1@126.com

^a^* Email: nanocarries@gmail.com

^d^* Email: minghua_md@fudan.edu.cn

^1.^These authors contributed equally to this work.


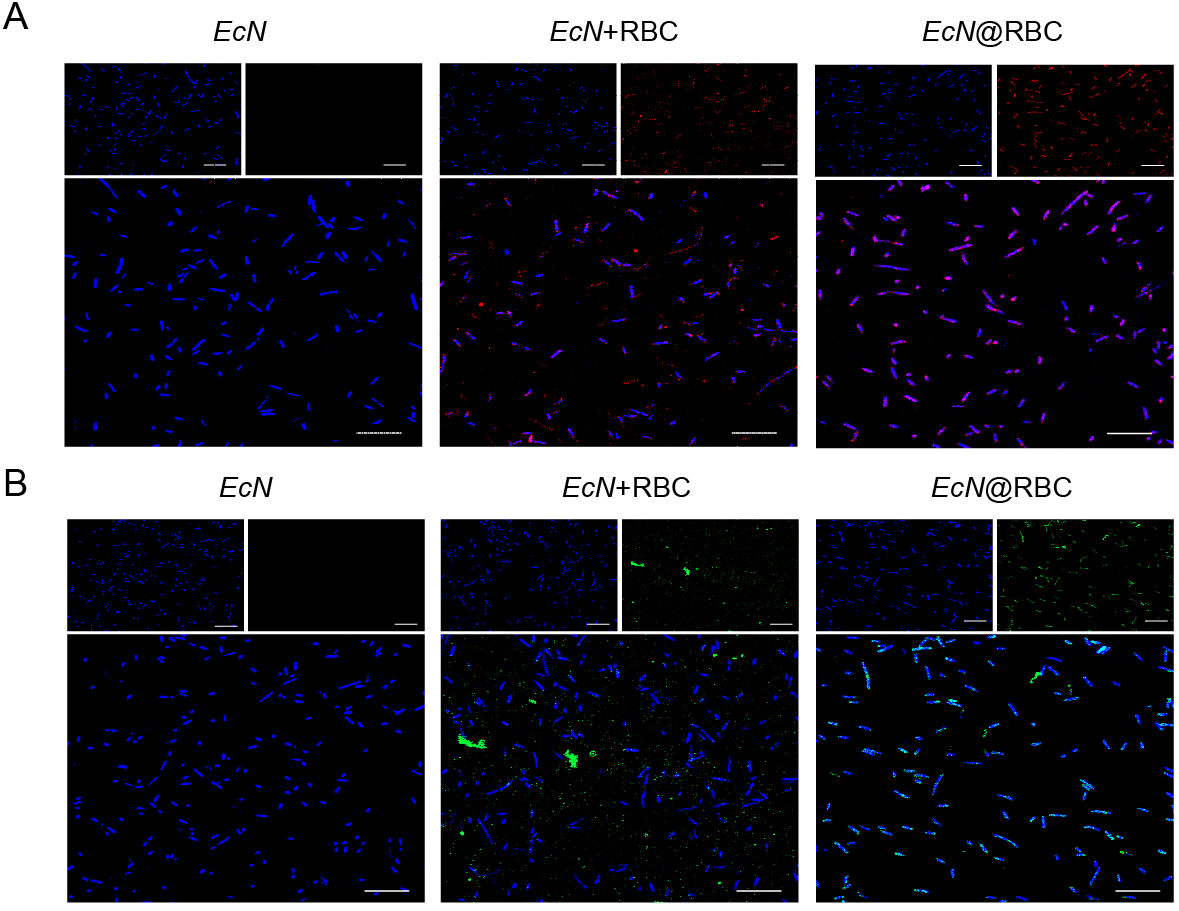


**Figure S1.** (A) CLSM images of *EcN*, *EcN*+RBC, and *EcN*@RBC. *EcN* was stained with blue (DAPI) and RBC membranes were stained with purple (DID), respectively (bar= 10 μm). (B) CLSM images of *EcN*, *EcN*+RBC, and *EcN*@RBC. *EcN* was stained with blue (DAPI) and CD47 was stained with green (FITC), respectively (bar= 10 μm).


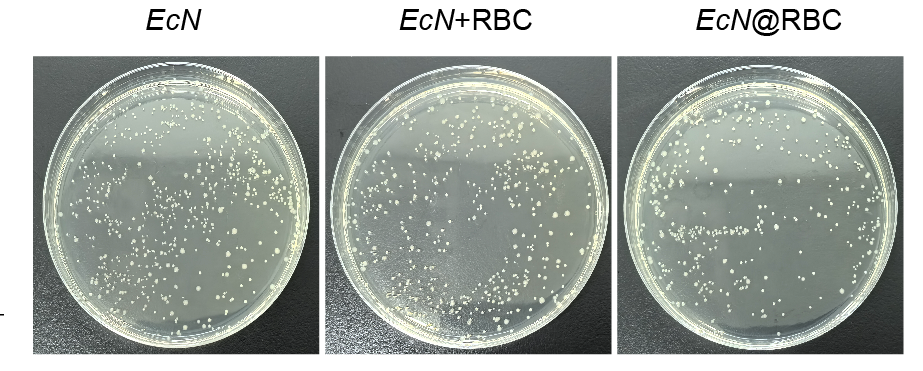


**Figure S2.** The colony pictures of *EcN*, *EcN*+RBC and *EcN*@RBC groups after 24 h (*EcN*, 1 × 10^6^ CFU/mL). .


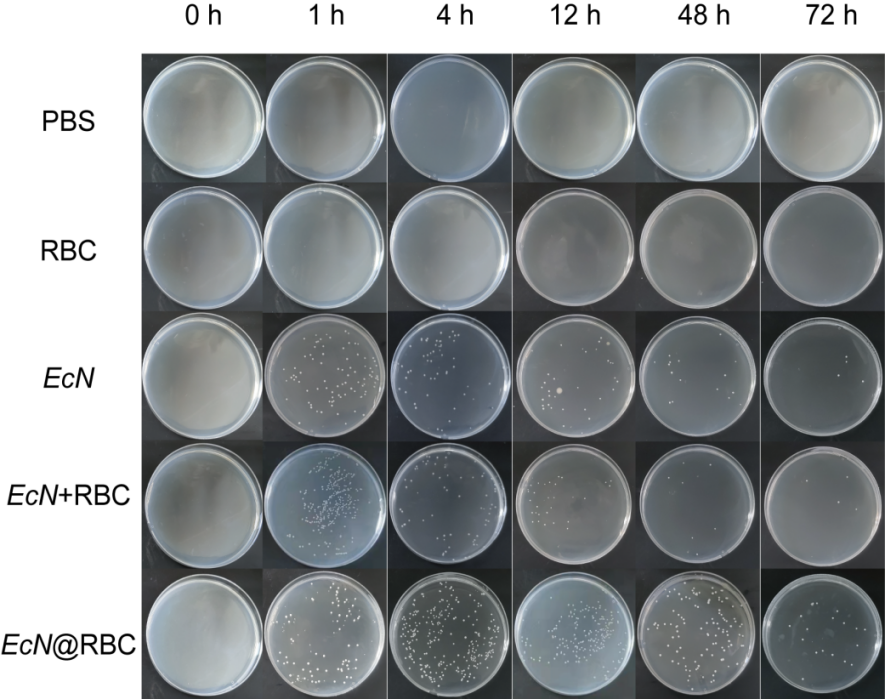


**Figure S3.** The representative photographs of solid LB agar plates of *EcN* colonization in blood harvested from CT26 tumor-bearing mice (n = 3).


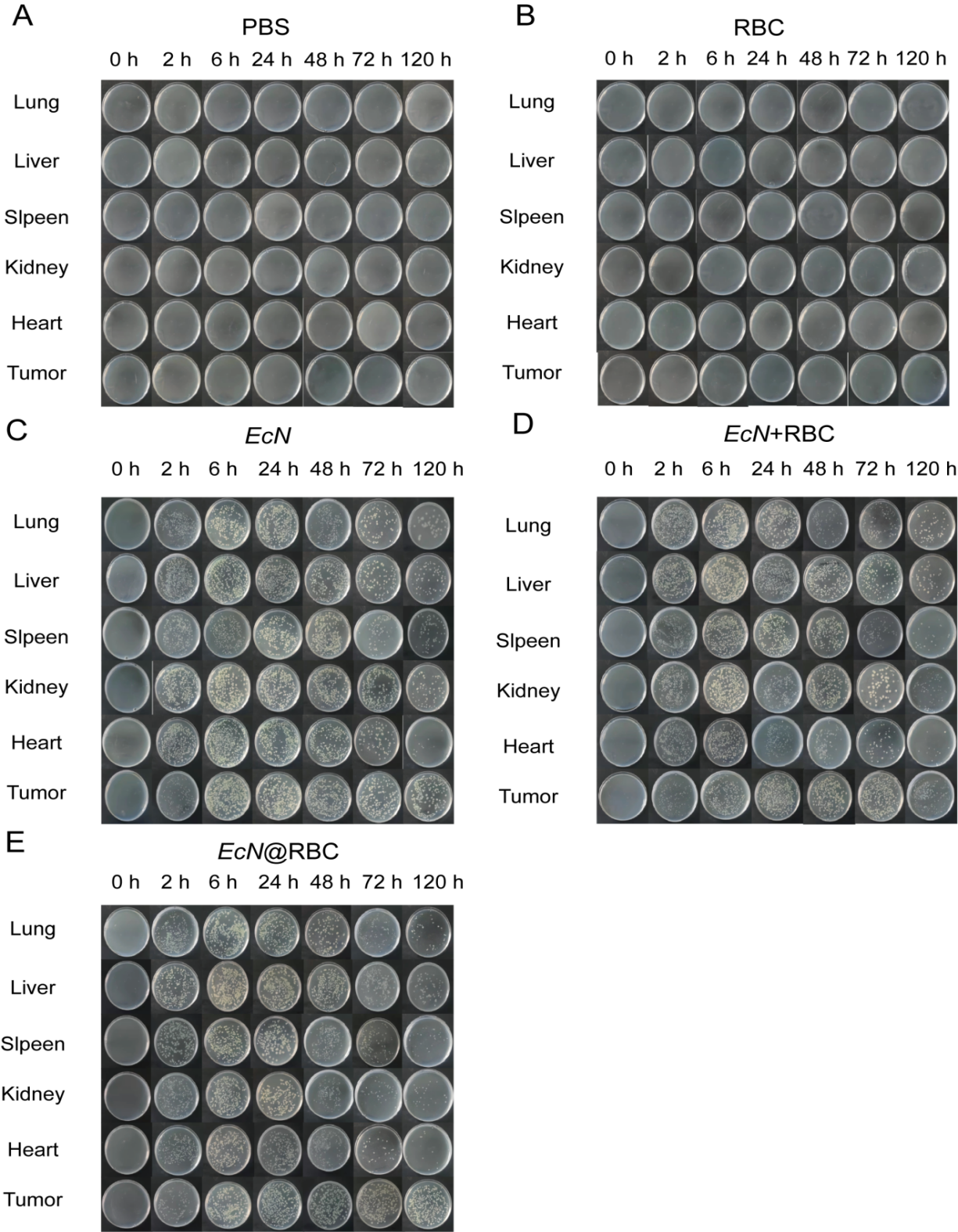


**Figure S4.** Biodistribution of bacteria in CT26 tumor-bearing mice. CT26 tumor-bearing mice were intravenously injected with A) PBS, B) RBC membrane, C) *EcN,* D) RBC*+EcN* and E) *EcN*@RBC *(EcN*, 1 × 10^6^ CFU/mL) and then sacriﬁced at different time points (0 h, 2 h, 6 h, 24 h, 48 h, 72 h and 120 h). Organ homogenates were diluted with PBS and incubated on LB agar plates at 37 °C for 24 h and counted. Biodistribution at 2 h, 6 h, 12 h, 24 h, 48 h, 72 h, 120 h, respectively (n = 3).


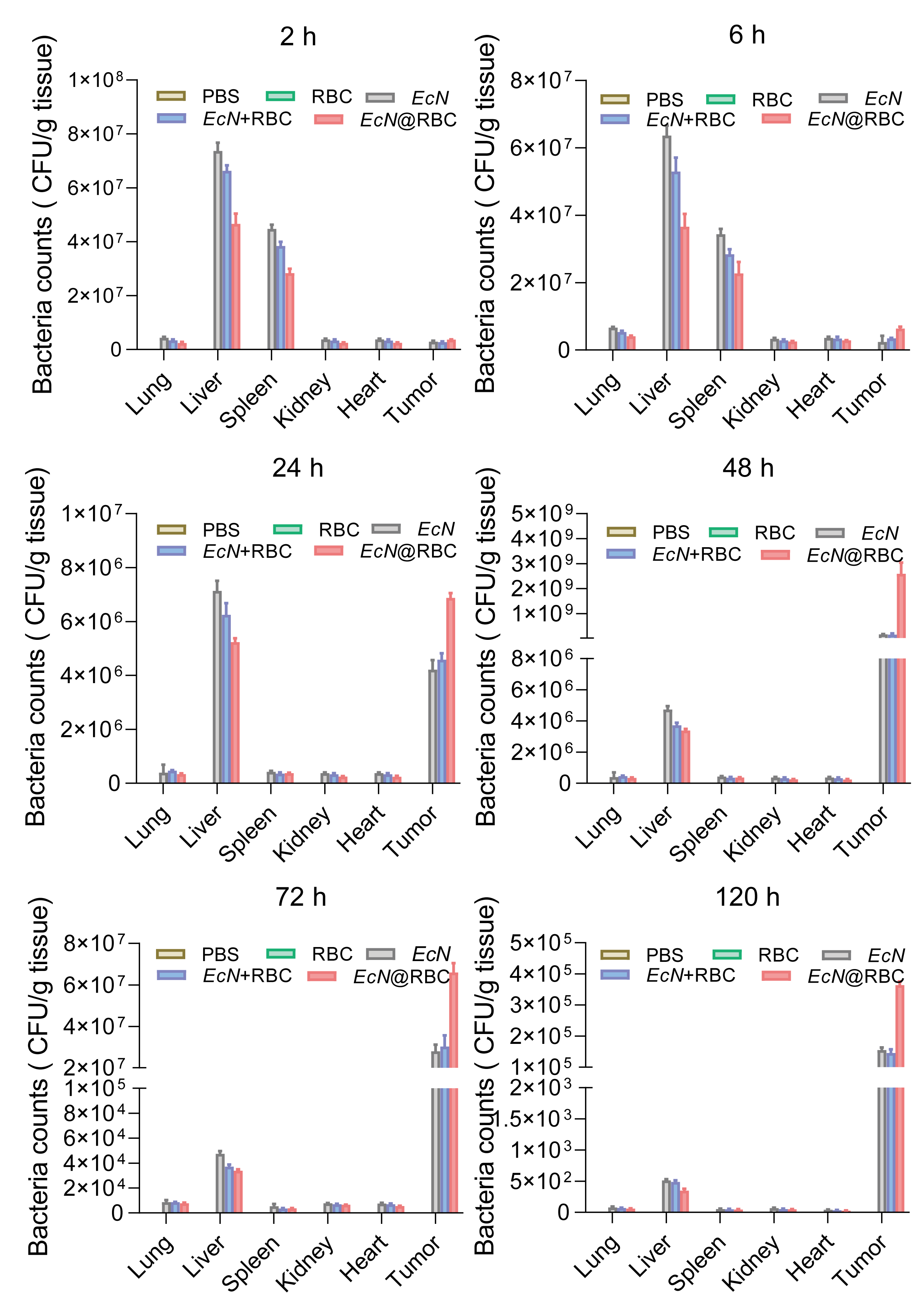


**Figure S5.** *In vivo* biodistribution of *EcN* in CT26 tumor-bearing mice. CT26 bearing Balb/c mice were intravenously injected with PBS, RBC membrane, *EcN*, RBC*+EcN* and *EcN*@RBC (*EcN*, 1 × 10^6^ CFU/ mL), then sacriﬁced at different time points (0 h, 2 h, 6 h, 24 h, 48 h, 72 h and 120 h). Heart, liver, spleen, lung and kidney tumor tissue were collected and homogenized at different time points. Tissue homogenates were diluted with PBS and incubated on LB agar plates for 24 h and counted. Biodistribution at A) 2 h, B) 6 h, C) 12 h, D) 24 h, E) 48 h, F) 72 h, G) 120 h, respectively (n = 3).


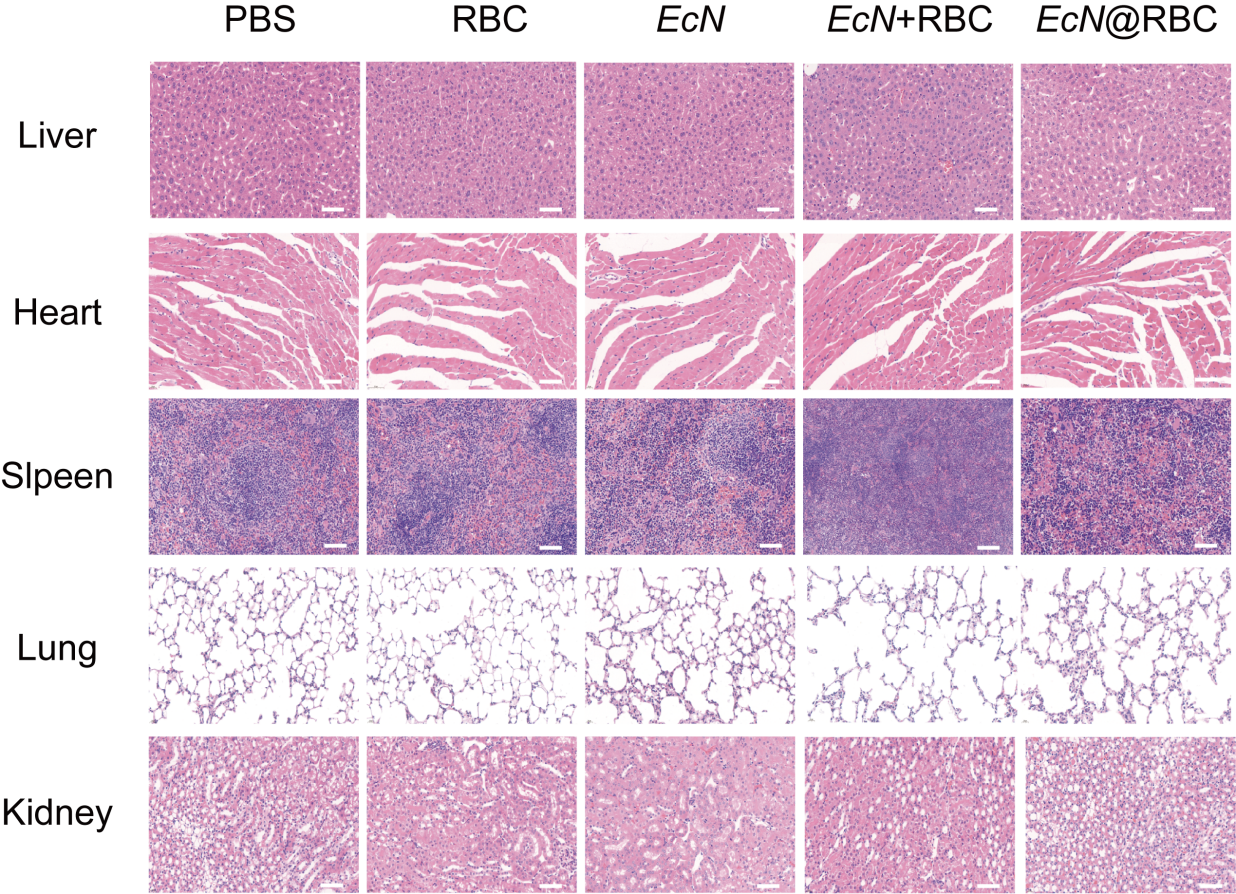


**Figure S6.** Representative images of H&E staining for major organs after treatments (Scale bar = 50 µm).


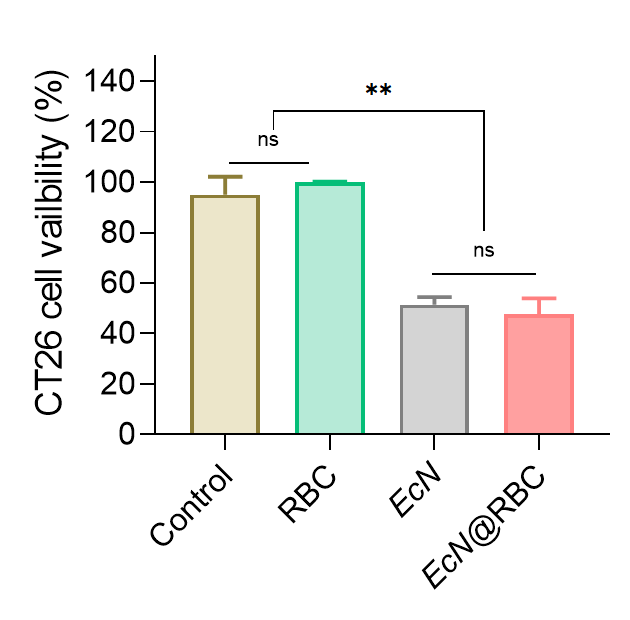


**Figure S7.** Relative cell viability of CT26 cells incubated with RBC, *EcN* and *EcN*@RBC (*EcN*, 1 × 10^5^ CFU/ml) for 6 h.


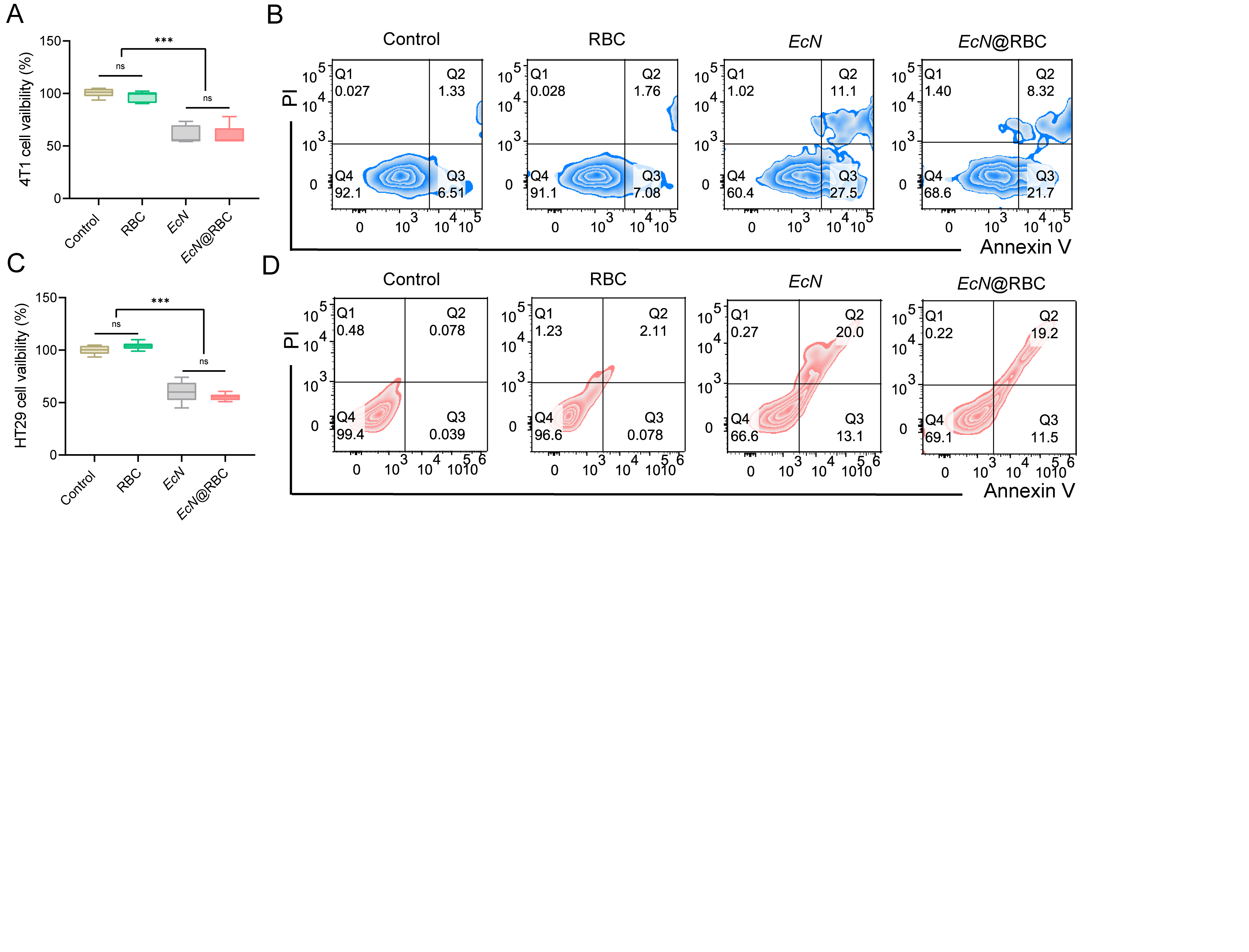


**Figure S8.** Relative cell viability of (A) 4T1 cells and (C) HT29 cells incubated with RBC, *EcN* and *EcN*@RBC (*EcN*, 1 × 10^5^ CFU/ml) for 6 h by CCK8. The apoptosis of (B) 4T1 cells and (D) HT29 cells by Flow cytometry analysis.

**
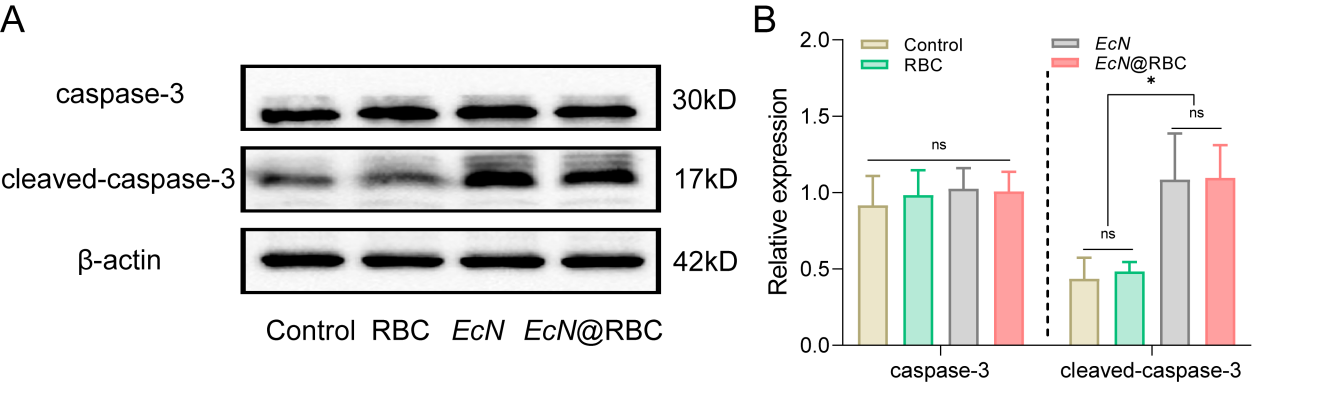
**

**Figure S9.** (A) The expression level of caspase-3 and cleaved-caspase-3 in different group. (B) The relative expression level of caspase-3 and cleaved-caspase-3 in different group. Significance was assessed using student's t-test, giving p values, *p < 0.05, ns indicates no statistical significance.


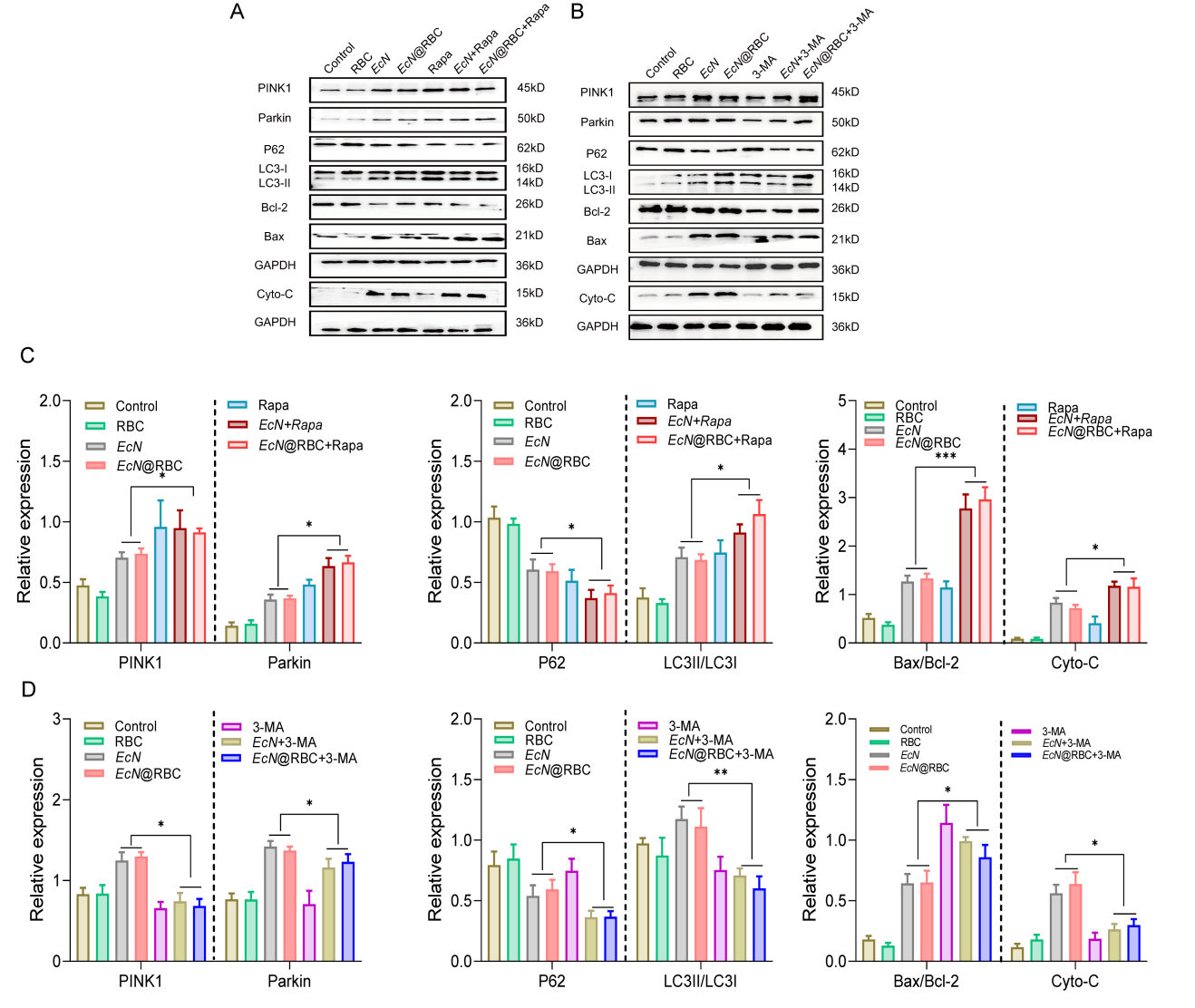


**Figure S10.** The related proteins expression under the treatment of (A) Rapa (50 µM) and (B) 3-MA (100 µM). (C)The relative expression level of PINK1, Parkin, P62, LC3, Bax/Bcl-2, and Cyto-C under the treatment of Rapa. (D)The relative expression level of PINK1, Parkin, P62, LC3, Bax/Bcl-2, and Cyto-C under the treatment of 3-MA. Significance was assessed using student's t-test, giving p values, *p < 0.05, **p < 0.01, ***p < 0.005.

**
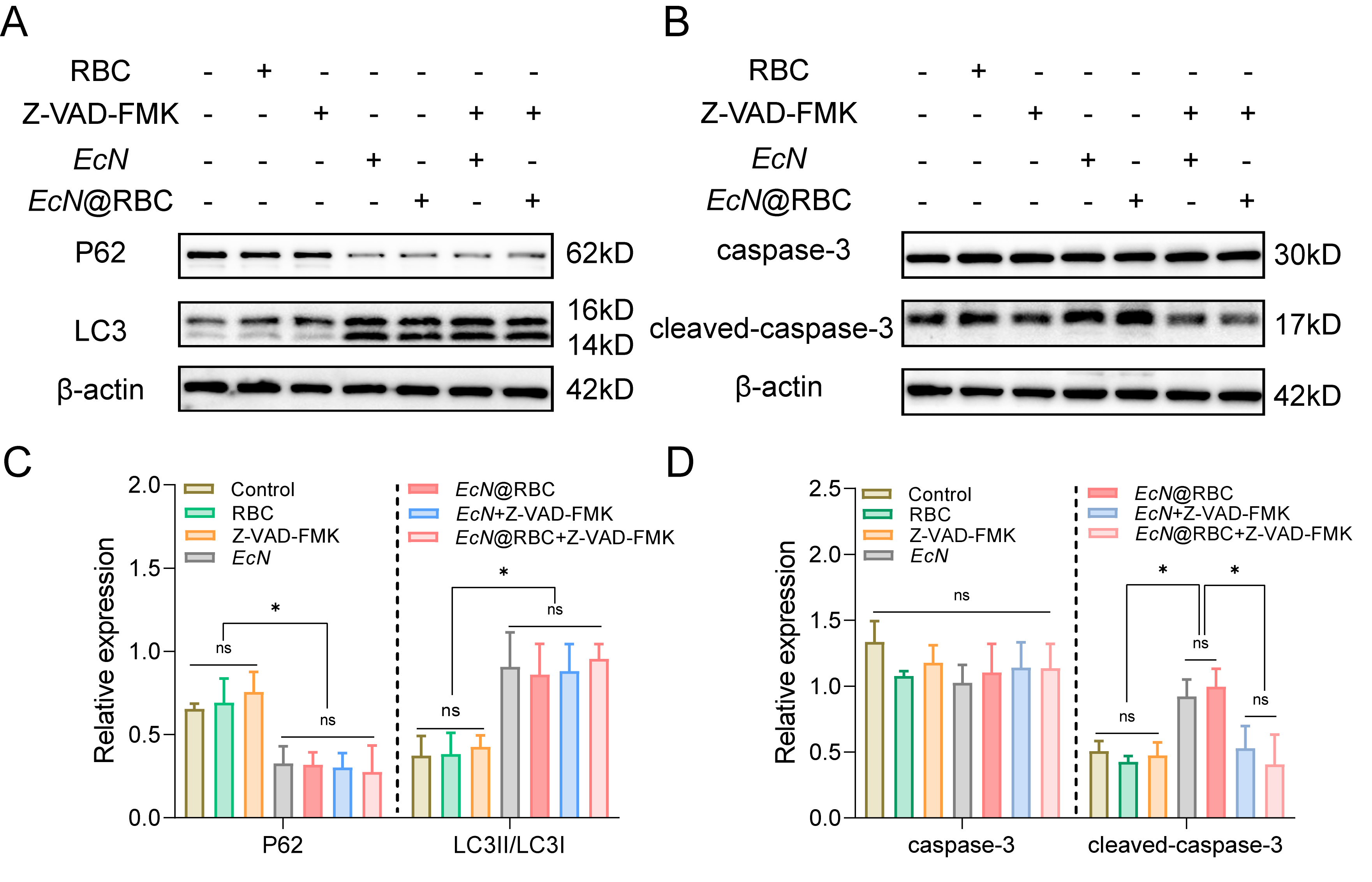
**

**Figure S11.** (A) The expression level of P62 and LC3 in different group. (B) The expression level of caspase3 and cleaved caspase3 in different group. (C) The relative expression level of P62 and LC3 in different group. (D) The relative expression level of caspase3 and cleaved caspase3 in different group.


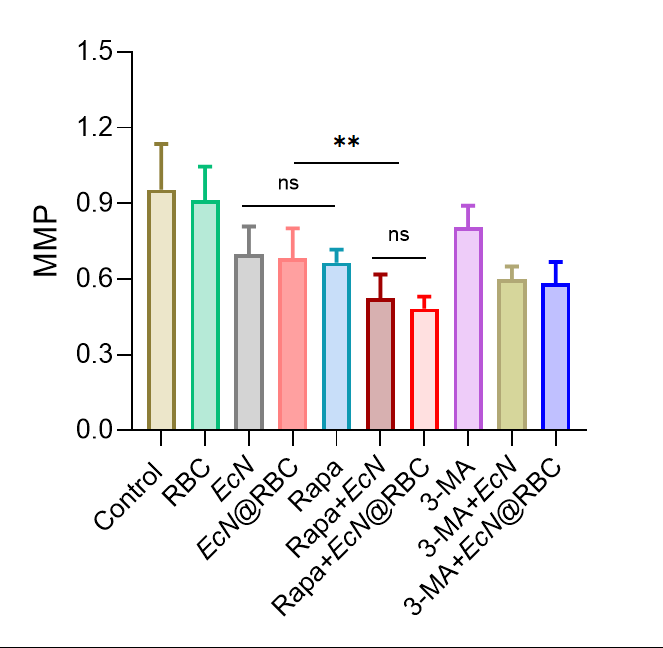


**Figure S12.** Mitochondrial membrane potential detection: fluorescence intensity in each group after treatment with Rapa and 3-MA were detected by fluorescent microplate reader.


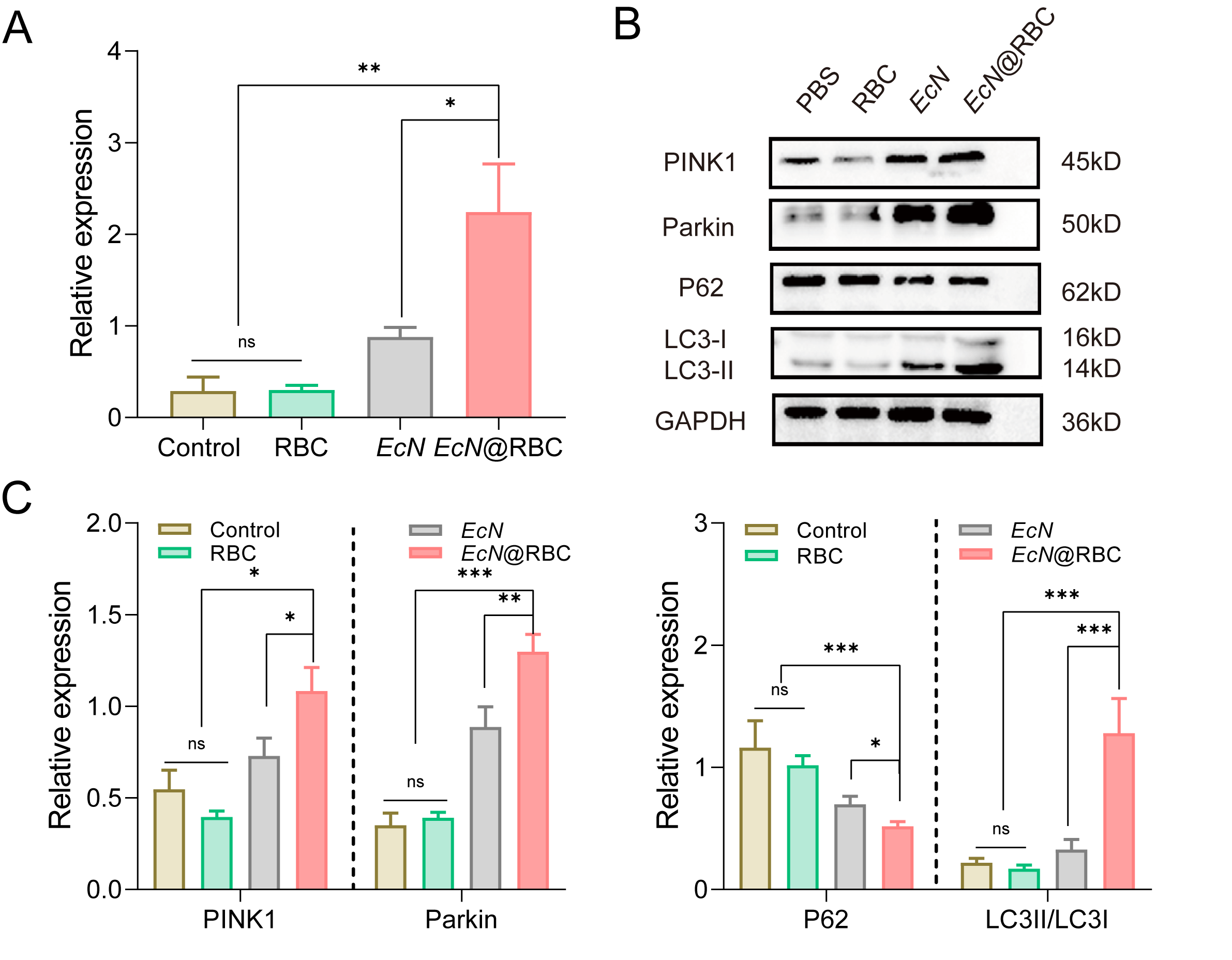


**Figure S13.** (A) The relative expression level of Bax/Bcl-2 in different group. (B) The expression level of PINK1, Parkin, P62, and LC3 in different group. (C) The relative expression levels of PINK1, Parkin, P62, and LC3 in different group.

**
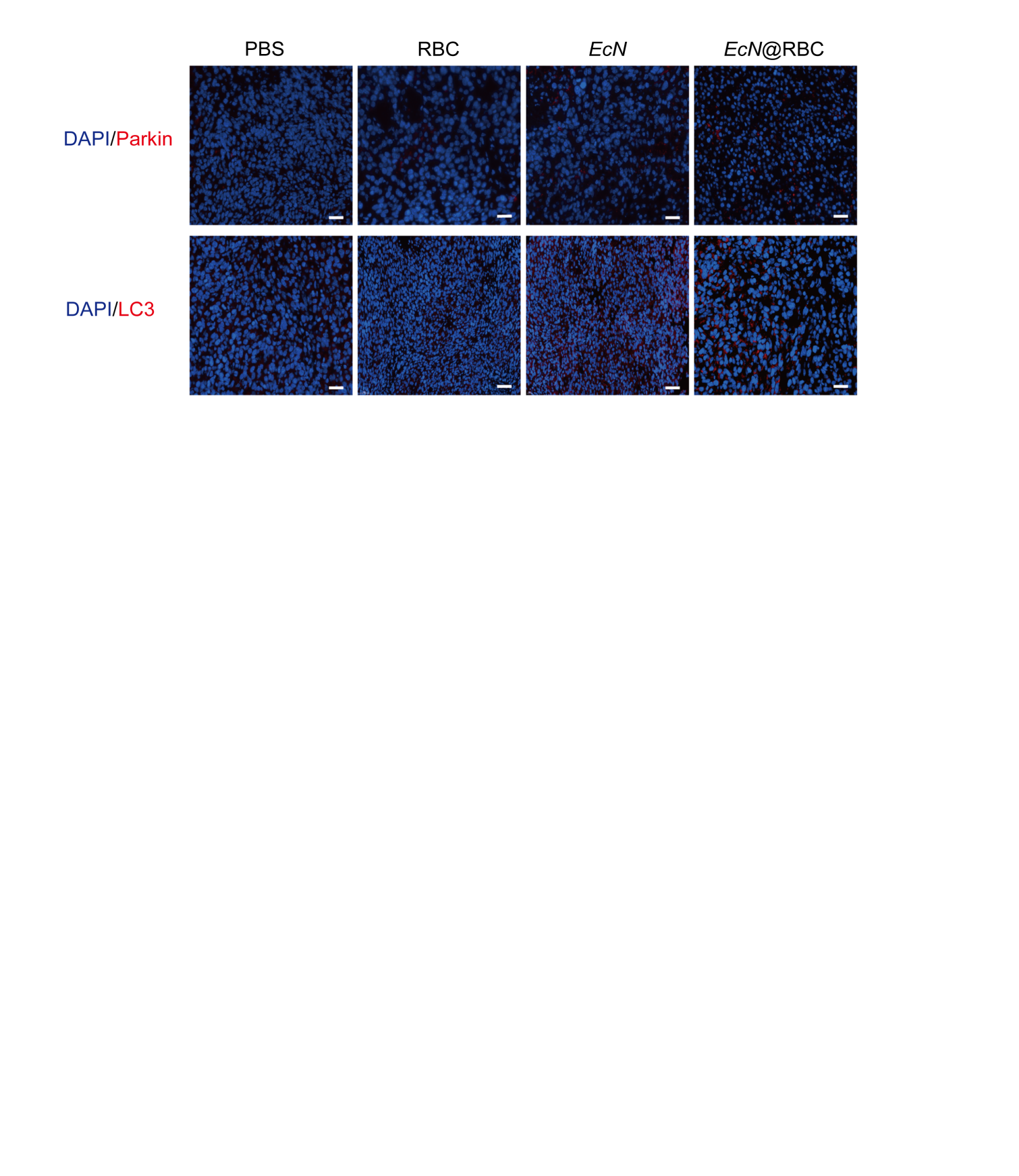
**

**Figure S14.** Representative immunofluorescence images of Parkin and LC3 expression in tumor tissue (Scale bar = 20 µm).

**
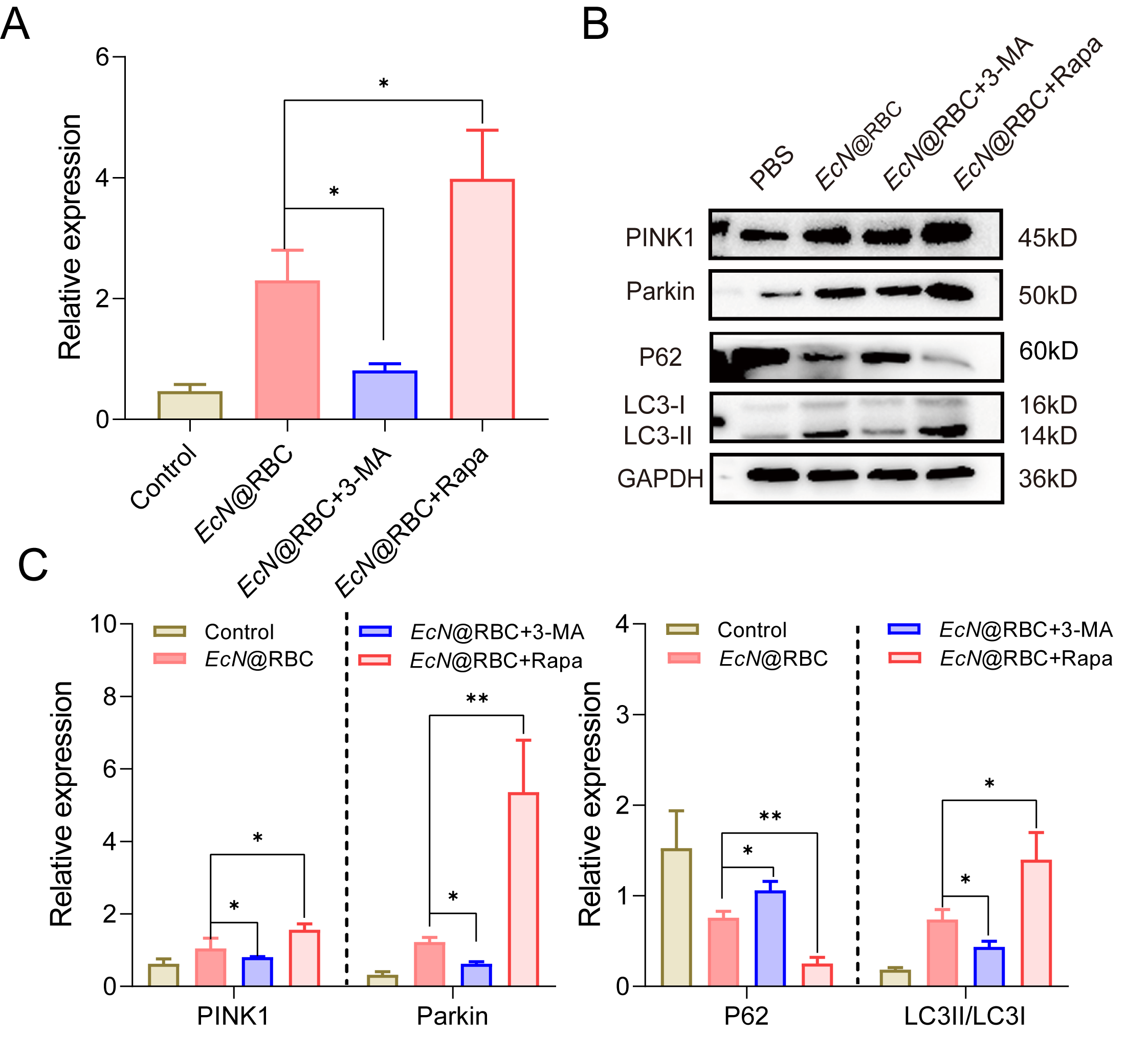
**

**Figure S15.** (A) The relative expression level of Bax/Bcl-2 in different group. (B) The expression level of PINK1, Parkin, P62, and LC3 in different group. (C) The relative expression levels of PINK1, Parkin, P62, and LC3 in different group.

**
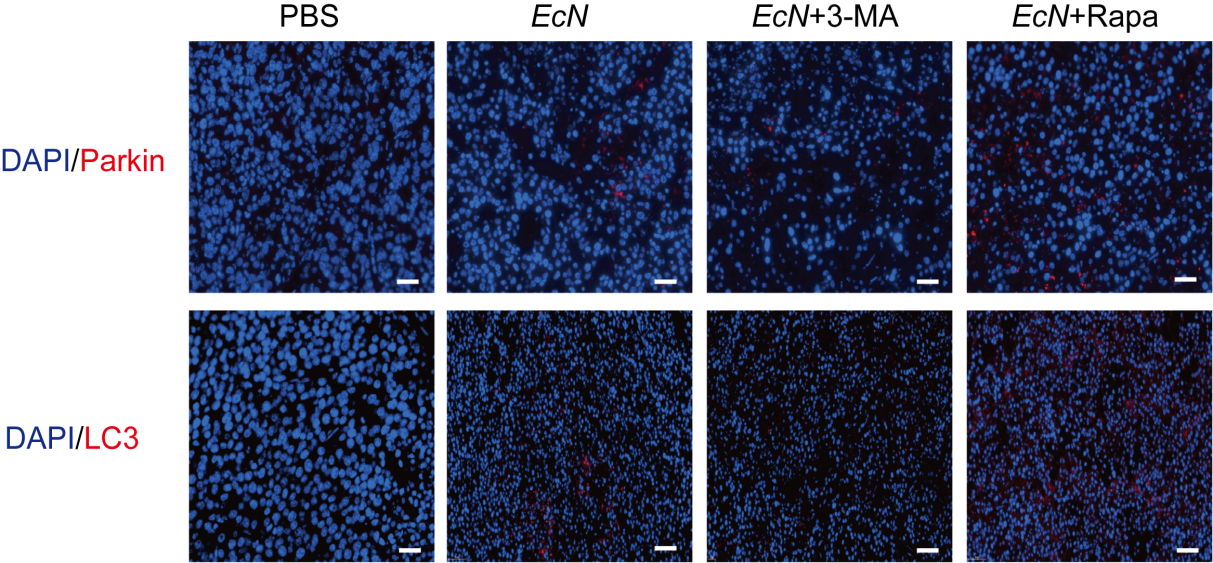
**

**Figure S16.** Representative immunofluorescence images of Parkin and LC3 expression in tumor tissue (Scale bar = 20 µm).
